# Gbdmr: identifying differentially methylated CpG regions in the human genome via generalized beta regressions

**DOI:** 10.1186/s12859-024-05711-y

**Published:** 2024-03-05

**Authors:** Chengzhou Wu, Xichen Mou, Hongmei Zhang

**Affiliations:** School of Public Health, University of Memphis, 3720 Alumni Ave, Memphis, TN 38152 USA

**Keywords:** Differentially methylated regions, Generalized beta distribution, DNA methylation, CpG site

## Abstract

**Background:**

DNA methylation is a biochemical process in which a methyl group is added to the cytosine-phosphate-guanine (CpG) site on DNA molecules without altering the DNA sequence. Multiple CpG sites in a certain genome region can be differentially methylated across phenotypes. Identifying these differentially methylated CpG regions (DMRs) associated with the phenotypes contributes to disease prediction and precision medicine development.

**Results:**

We propose a novel DMR detection algorithm, gbdmr. In contrast to existing methods under a linear regression framework, gbdmr assumes that DNA methylation levels follow a generalized beta distribution. We compare gbdmr to alternative approaches via simulations and real data analyses, including dmrff, a new DMR detection approach that shows promising performance among competitors, and the traditional EWAS that focuses on single CpG sites. Our simulations demonstrate that gbdmr is superior to the other two when the correlation between neighboring CpG sites is strong, while dmrff shows a higher power when the correlation is weak. We provide an explanation of these phenomena from a theoretical perspective. We further applied the three methods to multiple real DNA methylation datasets. One is from a birth cohort study undertaken on the Isle of Wight, United Kingdom, and the other two are from the Gene Expression Omnibus database repository. Overall, gbdmr identifies more DMR CpGs linked to phenotypes than dmrff, and the simulated results support the findings.

**Conclusions:**

Gbdmr is an innovative method for detecting DMRs based on generalized beta regression. It demonstrated notable advantages over dmrff and traditional EWAS, particularly when adjacent CpGs exhibited moderate to strong correlations. Our real data analyses and simulated findings highlight the reliability of gbdmr as a robust DMR detection tool. The gbdmr approach is accessible and implemented by R on GitHub: https://github.com/chengzhouwu/gbdmr.

**Supplementary Information:**

The online version contains supplementary material available at 10.1186/s12859-024-05711-y.

## Background

DNA methylation (DNAm), a biochemical procedure in which a methyl group is added to the cytosine, is a heritable epigenetic phenomenon. In mammals, DNAm mainly happens at the cytosine-phosphate-guanine (CpG) site. DNAm is critical in silencing retroviral elements, regulating tissue-specific gene expression, genomic imprinting, and X chromosome inactivation [1]. These processes influence the gene expression status and protein expressions, and they may be further involved in the development of diseases such as nervous disorders, cardiovascular diseases, and cancer [2–5].

DNAm is an important biomarker in the epigenetic study. CpG sites are methylated differently across phenotypes, including age, sex, and health conditions [6–8]. As DNAm plays a role in the biological pathways for various diseases, differentially methylated CpG sites help researchers identify risk factors of certain diseases and understand their underlying mechanisms. One classic strategy to study the association between CpG sites and phenotype is to regress each CpG’s DNAm levels on the phenotype through linear regression and screen for the most significant CpGs [9–11]. In this article, we refer to this approach as the traditional epigenome-wide association study (EWAS). However, phenotypes may be regulated jointly by CpGs in one region [12] or spatially close to each other. EWAS ignores such joint activities. Thus, a method that has the ability to identify differentially methylated regions (DMRs) fits the underlying biological mechanisms and is expected to achieve better performance.

A variety of DMR detection methods have been developed. A widely used idea is to apply meta-analysis-based approaches to synthesize or summarize single CpG sites’ summary statistics from the traditional EWAS. For example, dmrff [13] divides the candidate regions based on the physical distances between CpG sites and applies the traditional EWAS on each CpG site in the same region. Then, it uses the inverse-variance weighted meta-analysis method to combine the EWAS outputs and searches for the most statistically significant sub-regions. Comb-p [14] starts from the EWAS *p*-values. It estimates spatial auto-correlation at different distance lags and adjusts *p*-values for adjacent CpG sites based on the Stouffer-Liptak-Kechris correction. The regions are determined based on the adjusted *p*-values, and the *p*-value of each region is re-calculated based on the Stouffer-Liptak-Kechris correction and auto-correlation function again. DMRcate [15] uses a Gaussian kernel smoother to adjust the *p*-values of the EWAS results for each CpG site. Then, the statistical significance is re-calculated based on the smoothed t-statistics, and the significant CpG sites within a specific distance are treated as DMRs. Similar to these methods, GlobalIP [16] also starts from the traditional EWAS outputs and uses the covariance matrix in the test statistic to account for the partial correlation among the CpG sites. In addition to the idea of summarizing EWAS results, seqlm [17] applies linear mixed models to segment CpG regions and fit the DNAm data simultaneously. Lent et al. [18] conducted a comprehensive comparison among the aforementioned methods, in which dmrff was shown to have the highest power in most settings. Following the findings of Lent et al., in this article, we set dmrff as the benchmark of DMR detection algorithms to evaluate the proposed method.

Overall, both meta-analysis-based (e.g., dmrff) and model-based (e.g., seqlm) methods are restricted to the underlying framework of linear regression between DNAm level and phenotype. This framework assumes DNAm levels follow a normal distribution. However, the normality of DNAm data is questionable. Generated by Illumina's 450k BeadChip or EPIC array, DNAm is calculated as max(|*M*|,0)/(|*M*| + |*U*| + *c*), denoted beta value with *M* and *U* being the intensities of methylated and unmethylated probes; *c* is a positive constant value to regularize the DNAm value when both *M* and *U* are too small [19]. Beta value is thus a ratio that ranges from 0 to 1, and it is natural to model beta values via beta distribution families. To meet the normality assumption, researchers often take logit transformation to convert a beta value to an M value. However, the normality of M value through such a transformation is problematic [20].

In this paper, we propose an innovative model-based DMR detection approach, generalized beta differentiated methylation region (gbdmr) detection method. Unlike conventional approaches that assume a normal distribution for the response variable, gbdmr employs Generalized Beta Regression that models multiple adjacent CpG sites jointly as ratios. To be specific, the gbdmr segments the candidate regions by physical coordinates and correlation patterns. It then uses the generalized beta distribution to straightforwardly model the DNAm of CpG sites in each region and calculates the corresponding *p*-values. The *p*-value for each region reflects statistical association significance between a phenotype and DNAm. Generalized beta distribution fits the definition of DNAm defined as beta values, and in addition, accounts for the correlation structures among adjacent CpG sites. It has been used to model the distribution of nominal family income and stock indexes in economics [21] and to identify the intergenerational patterns of DNAm levels [22] in the epigenetic study.

To assess the performance of the proposed method, we conducted simulation studies and real data analysis using gbdmr, dmrff, and EWAS. In simulation studies, gbdmr achieved higher power than dmrff when the correlations between adjacent CpGs were high; on the contrary, the power of dmrff is higher when the correlations are weaker. We proved the performance decay of dmrff as the correlation increases theoretically, and postulate this phenomenon may be universe among meta-analysis-based methods. The gbdmr has been implemented in the R software. The R package is available through GitHub: https://github.com/chengzhouwu/gbdmr.

The remaining article is structured as follows. The Method section includes the detailed steps of gbdmr. The Results section presents the simulation results and real data analysis. The Materials section provides technical details of simulation settings and real data analysis. In the Discussion section, we explain the possible reasons for the performance disparity between gbdmr, dmrff, and other DMR detection methods under different correlations of adjacent CpG sites. The Conclusion section summarizes the key findings and the implications. We include the theoretical proof, additional simulation and data analysis results in the Additional File 1.

## Methods

The gbdmr consists of three key steps as stated below.

### Segment CpG sites into blocks

**Fig. 1:**
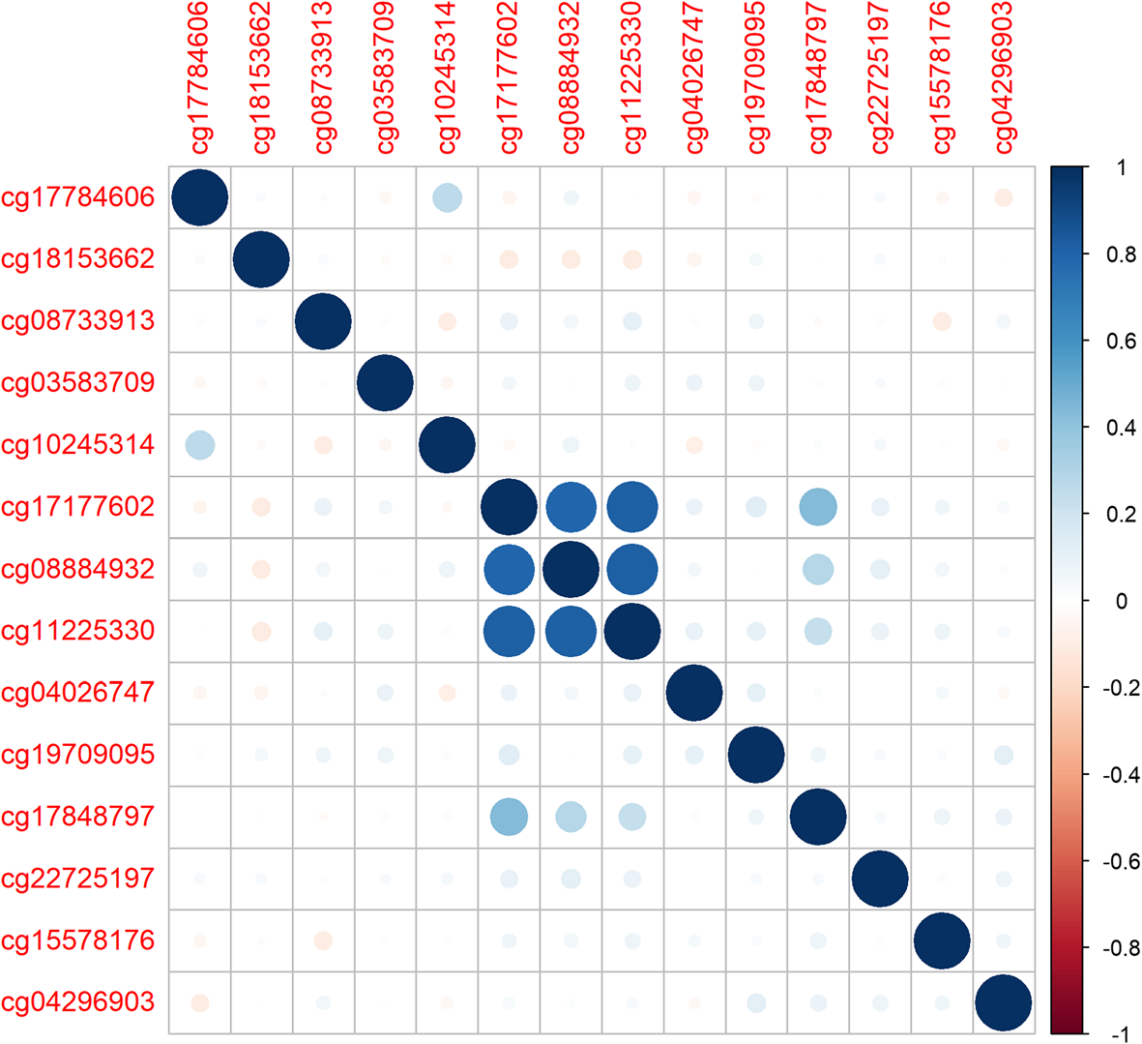
Correlation heatmap for a segment of adjacent CpGs. The labels on the x and y axes are CpG names assigned by Illumina

CpG sites are more likely to be correlated when their physical locations are close to each other [12, 23, 24]. We implement a strategy to identify highly correlated regions: CpG sites are ordered by the chromosome numbers and chromosome coordinates. In each chromosome, adjacent CpG sites' Pearson correlation is calculated one by one. A chain of CpG sites forms a block if the correlation of each pair of neighboring CpG sites is higher than a certain threshold. We suggest a threshold of 0.5, i.e., consecutive CpG sites with neighboring correlation stronger than 0.5 form a block. If a CpG site does not have a sufficiently strong correlation with either of its neighbors, it forms a block by itself. For example, Fig. 1 presents the correlation heatmap of a segment of adjacent CpGs in chromosome 1. We identify the sites and region based on the proposed strategy: The correlations of cg17177602 and cg08884932, cg08884932 and cg11225330 are larger than 0.5, which forms a block of three consecutive CpG sites. The other CpGs do not have a strong enough correlation with their neighbors. Thus, each of them forms a block consisting of a single CpG site.

### Model the blocks

Suppose we divide *m* consecutive CpG sites into *B* blocks following the strategy of block segmentation. Denote $L_b$ the block size, i.e., number of CpG sites in the *b*th block, where *b* = 1, ..., *B* and $\sum _{b=1}^B L_b=m$. When $L_b=1$, the *b*th block is a single CpG site; when $L_b \ge 2$, the *b*th block is a region containing multiple CpG sites. We use generalized beta distribution to model the DNAm level(s) of CpG site(s) in each block.

In the *b*th block, denote $\pmb {Z}_b=(Z_{1b}, \dots , Z_{L_bb})$, DNAm levels of the $L_b$ CpG sites. Following Libby and Novick [25], we define $\pmb {Z}_b$ follows a $L_b$-variate generalized beta distribution, denoted by $Gbeta(\pmb {\alpha }_b, \beta _b)$, if $Z_{lb}=P_{lb}/(P_{lb}+Q_b)$ for $l=1,\dots ,L_b$, where $P_{lb} \sim Gamma(\alpha _{lb},1)$, $Q_b \sim Gamma(\beta _b,1)$, $P_{lb}$’s and $Q_b$ are independent, and $\pmb {\alpha }_b$ = $(\alpha _{1b}, \dots , \alpha _{L_bb})$. Here $\alpha _{lb}>0$ for $l=1,\dots ,L_b$ and $\beta _b>0$. The density function $f(\pmb {Z}_b| \pmb {\alpha }_b,\beta _b)$ can be expressed as

1 $$f(\pmb {Z}_b| \pmb {\alpha }_b,\beta _b) = \frac{{\Gamma \left( {\sum\limits_{{l = 1}}^{{L_{b} }} {\alpha _{{lb}} } + \beta _{b} } \right)\prod\limits_{{l = 1}}^{{L_{b} }} {\left\{ {\left( {\frac{{Z_{{lb}} }}{{1 - Z_{{lb}} }}} \right)^{{\alpha _{{lb}} - 1}} \left( {\frac{1}{{1 - Z_{{lb}} }}} \right)^{2} } \right\}} }}{{\Gamma (\beta _{b} )\prod\limits_{{l = 1}}^{{L_{b} }} \Gamma (\alpha _{{lb}} )\left\{ {1 + \sum\limits_{{l = 1}}^{{L_{b} }} {\left( {\frac{{Z_{{lb}} }}{{1 - Z_{{lb}} }}} \right)} } \right\}^{{\sum\limits_{{l = 1}}^{{L_{b} }} {\alpha _{{lb}} } + \beta _{b} }} }}$$

Note that when $L_b=1$, i.e, the block contains one single CpG site, the generalized beta distribution is trivialized to a univariate beta distribution. When $L_b \ge 2$, the marginal distribution of each CpG site also follows a univariate beta distribution. Thus, the generalized beta distribution has the ability to model each CpG with an ordinary beta distribution, which fits the definition of the beta value of a CpG site. Through the shared $Q_b$ in the definition, a generalized beta distribution incorporates correlation among CpG sites. This feature enables us to model correlation structures among adjacent CpG sites.

Now, we link the expected value of DNAm levels to the phenotype of interest through generalized beta regression. Denote $\pmb {Z}_b^i=(Z^i_{1b},\dots , Z^i_{L_bb})$ the DNAm levels of the *b*th block for the *i*th sample, *i* = 1,..., n. We assume $\pmb {Z}_b^i \sim Gbeta(\pmb {\alpha }^i_b,\beta ^i_b)$, where $\pmb {\alpha }^i_b=(\alpha ^i_{1b},\dots ,\alpha ^i_{L_bb})$. Denote $\pmb {X}^i=(1,X_1^i,\dots ,X_p^i)^\top$ a vector of independent variables for sample *i*. Here 1 is for the intercept; $X_1^i$ is the phenotype of interest for the *i*th sample; $X_1^i$ could be binary (e.g., sex) or continuous (e.g., age); $X^i_{2},\dots ,X^i_{p}$ are the $p-1$ covariates or confounders that need to be adjusted for. We build a logit function to link the independent variables to the mean of $Z^i_{lb}$ for $l=1,\dots ,L_b$ as below.

2 $$\begin{aligned} \text{ logit }\{E(Z^i_{lb})\}&= \text{ logit }(\frac{\alpha ^i_{lb}}{\alpha ^i_{lb} + \beta ^i_b}) \nonumber \\&= \log (\alpha ^i_{lb}) - \log (\beta ^i_b) \nonumber \\&= \pmb {X}^{i\top }\pmb {\gamma }_{lb}, \end{aligned}$$

where $\pmb {\gamma }_{lb}=(\gamma _{0lb},\gamma _{1b},\dots ,\gamma _{pb})^\top$, in which $\gamma _{0lb}$ denotes the CpG-specific intercept for the *l*th CpG in the *b*th block, and $\gamma _{1b},\dots ,\gamma _{pb}$ denote the block-specific coefficients of independent variables for the *b*th block. Note that $\gamma _{1b}$ is of our interest indicating the effect of variable $X_1$ on DNAm in block *b*.

### Apply likelihood ratio test

Combining the density function (1) and Eq. (2), we can obtain the likelihood function $L(\pmb {\theta }_b)=\prod _{i=1}^{n}f(\pmb {Z}^i_b|\pmb {\theta }_b,\pmb {X}^i)$ for the *b*th block. Here *n* is the sample size, and we reparameterize the parameters $(\pmb {\alpha }_b,\beta _b)$ to $\pmb {\theta }_b=(\pmb {\gamma }_{lb},\beta _b)$ based on Eq. (2). To apply the likelihood ratio test, we calculate the difference of two log-likelihoods $2\ln \{\sup _{\pmb {\theta }_b\in \pmb {\Theta }}L(\pmb {\theta }_b) \}-2\ln \{\sup _{\pmb {\theta }_b\in \pmb {\Theta }_0}L(\pmb {\theta }_b)\}$, where $\pmb {\Theta }$ denotes the whole parameter space for $\pmb {\theta }_b$, and $\pmb {\Theta }_0$ denotes the subspace constrained by the null hypothesis $\gamma _{1b=0}$. This difference follows a chi-square distribution with a degree of freedom 1. We apply the likelihood ratio test to obtain the *p*-value for each block. In the end, all *p*-values will be adjusted for multiple testing across the blocks. The statistically significant blocks are identified as DMRs.

## Results

The performance of gbdmr was evaluated through both simulation studies and real data analyses. In a recent study that compared various DMR detection methods, including DMRcate, comb-p, seqlm, GlobaIP, and dmrff [18], it was found that dmrff had the highest power in most settings while maintaining a low false positive rate. Therefore, dmrff was chosen as the benchmark DMR detection method. Additionally, we include the traditional EWAS, which did not consider correlation structures, as a comparison approach to assess the benefit of DMR in detecting potentially informative CpGs. It is important to note that different methods for DMRs may not identify the exact same regions even if they are in close proximity. For instance, one method may detect CpG sites 1–3 as a DMR, while another method may detect CpG sites 2–6 as a DMR. Hence, using the number of DMRs alone is not an appropriate method to evaluate the performance of different detection methods. Instead, a more accurate approach is to consider the number of **DMR CpGs**, which refers to the total number of CpG sites within the positive DMRs identified by the method. Additionally, isolated single CpG sites that show differential methylation are referred to as differentially methylated positions (DMPs). We refer to these CpG sites as **DMP CpGs** in this paper.

### Simulations

Simulations were used to evaluate how (1) the strength of correlations among adjacent CpG sites, (2) block size, and (3) effect size influence the performance of the three methods. We simulated DNAm data associated with a binary phenotype. Technical details are included in Sect. “Simulation method”.

**Fig. 2:**
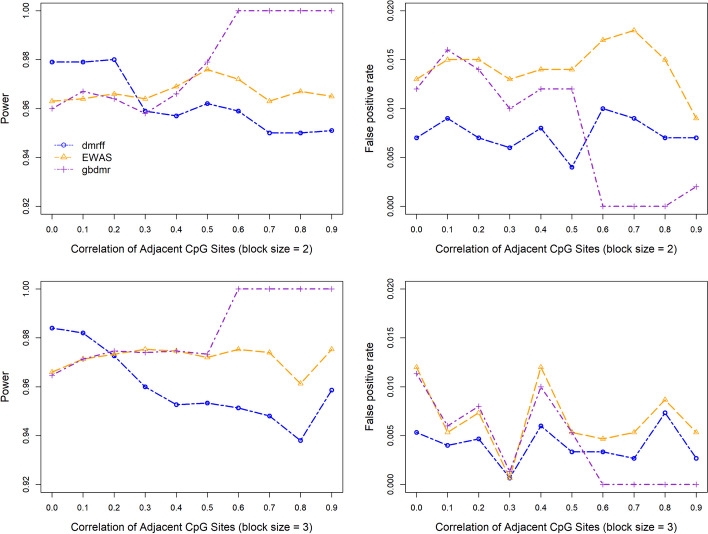
Power and false positive rates of gbdmr, dmrff, and EWAS across different block sizes

Figure 2 illustrates the power (sensitivity) and false positive rate (1 - specificity) across different correlation strengths of adjacent CpG sites, denoted by $\rho$, and block sizes. The power of all methods is calculated as the number of DMR CpGs divided by the total number of CpGs. When block size $=2$, the power of traditional EWAS stays at a relatively stable level. Gbdmr performs very similarly to EWAS when $\rho \le$ the threshold 0.5. In this case, the correlation between the two adjacent CpG sites is not strong; we treat each as an independent CpG site and fit univariate beta regression on each CpG separately. We observe that the performance of univariate beta regression on single CpG sites is very close to that of EWAS which utilizes linear regressions. When $\rho >0.5$, however, the power of gbdmr reaches almost 1, much higher than EWAS. It indicates that the gbdmr may perform better for strongly correlated CpG sites. In comparison with dmrff, we observe that dmrff has a higher power than gbdmr and EWAS when the correlation between adjacent CpG sites is low, while the power decreases as the correlation increases. We provide a theoretical explanation of this phenomenon in Sect. “Discussion”. As for specificity, all three methods keep the false positive rate at a low level. The patterns of the three methods for block size $=3$ are similar to those of block size $=2$. In Additional file 1: Appendix A.1, we visualized the frequency of block sizes in the three datasets of our real data analysis utilizing histogram. We found more than 99.9% of block sizes range between 1 and 10, and over 99.8% of block sizes are less than 6. To reflect these block sizes in the real data and examine the robustness of gbdmr, we further applied a range of settings to compare the three methods, such as block sizes 4–10 and different correlation thresholds of $\rho$. The details of the extended simulations are in Additional file 1: Appendices A.2 and A.3. In general, the simulation results are consistent with the findings presented in Figs. 2.

**Fig. 3:**
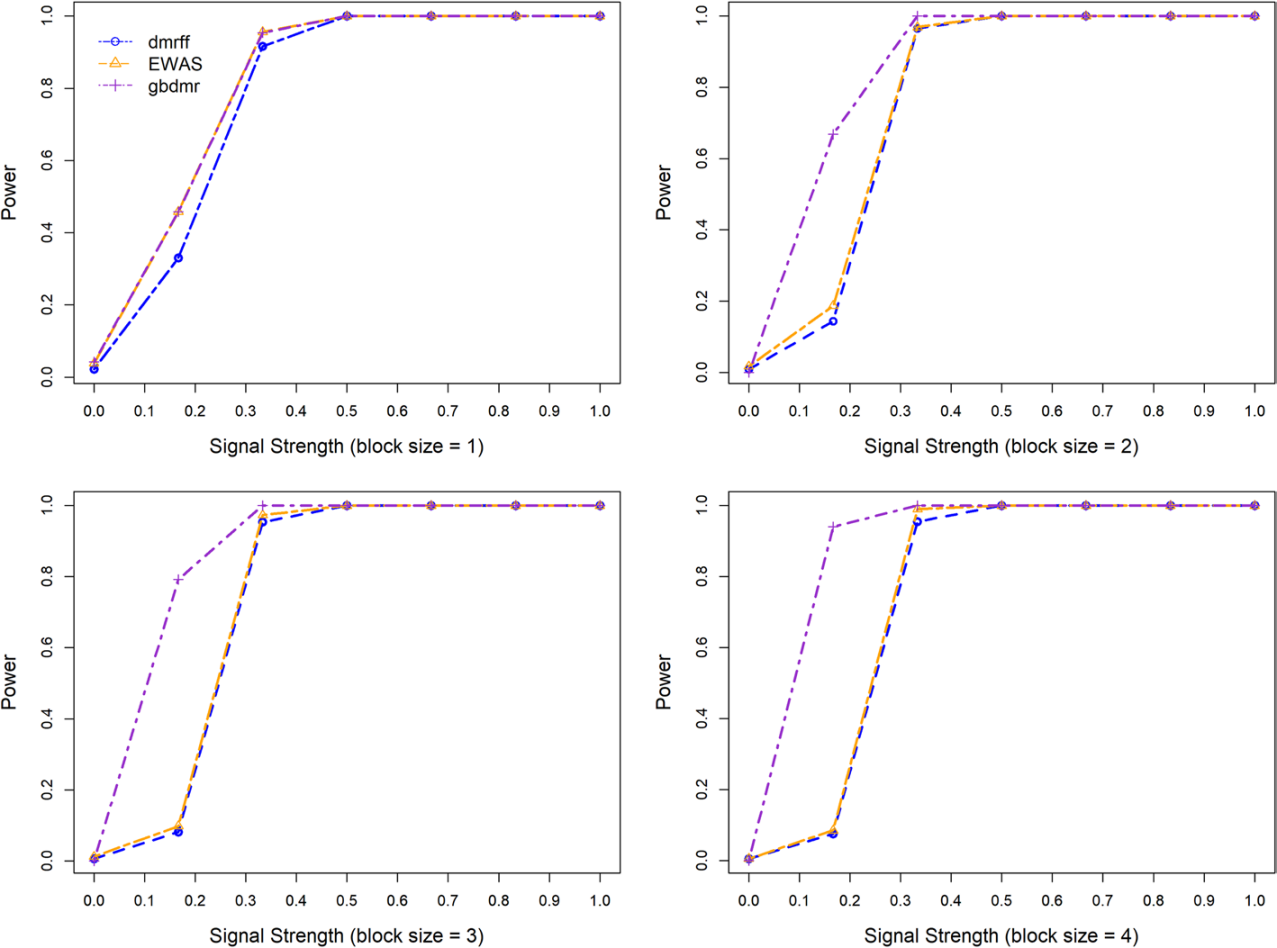
Power over different signal strengths of gbdmr, dmrff, and EWAS across different block sizes

Figure 3 shows how power changes with signal strength when block sizes $=1,2,3,$ and 4. The $\rho$ is set to be 0.8 when the block size is larger than one. The signal strength in Fig. 3 is defined by the difference of means in DNAm between the phenotype presence and absence groups divided by the standard deviation. The power is calculated as the number of positive DMP/DMR CpGs divided by the total number of CpGs when the block size equals/larger than 1. Note that when signal strength=0, i.e., the true distributions of the two groups are the same, the y-axis is a false positive rate instead of power. When block size $= 1$, gbdmr works as well as EWAS, and both have slightly higher power than dmrff. For block size of 2, when signal strength $=0$, i.e., the true distribution of the two phenotype groups are the same, all three methods identify no significant CpG sites, indicating a perfect specificity. When the signal strength lies between 0 and approximately 0.5, gbdmr has uniformly higher power than the other two methods. When the signal strength $\ge 0.5$, the power of all three methods reaches one. Similar patterns are found when block size $=3$ and 4. Various settings including larger block sizes are utilized to assess the performance of the three methods; these extended simulation results are presented in Additional file 1: Appendix B, in which gbdmr consistently exhibits greater statistical powers compared to both EWAS and dmrff.

In summary, gbdmr performs similarly to EWAS when the correlation between adjacent CpGs $\le$ the threshold 0.5. Gbdmr is superior to the other two methods when CpG sites are highly correlated. On the contrary, dmrff shows a higher power when the correlation is weak, and the power decreases as the correlation grows stronger.

### Real data analysis

We further applied the three methods to identify DMRs and DMPs in three DNA methylation datasets. One dataset is from a cohort study conducted on the Isle of Wight (IOW), United Kingdom[26], while the other two are from the Gene Expression Omnibus database repository (GSE) and focused on age-related DNA methylation profiles[27, 28]. We screened for DMRs and DMPs associated with various phenotypes, including age, sex, gestational age, and birth weight, and presented the number of identified DMRs and DMPs in Table 1. In total, ten analyses were conducted.

Table 1 shows that gbdmr outperformed dmrff in identifying DMR CpGs in most scenarios. Specifically, gbdmr detected more DMR CpGs than dmrff in eight out of ten analyses. Moreover, in eight out of ten analyses, the number of DMP CpGs identified by gbdmr was slightly higher than that by dmrff. In addition, both DMR detection methods outperformed EWAS in identifying a greater number of CpG sites (DMP CpGs $+$ DMR CpGs).

Consistent with the simulation study in Fig. 2, gbdmr is more sensitive than dmrff in identifying highly correlated DMRs in the real data analysis. Due to the block segmentation algorithm, gbdmr only identifies DMRs with neighboring DMR CpG’s correlation $\rho$ higher than the pre-specified threshold. In Table 1, compared to dmrff, gbdmr detects more DMR CpGs in most analyses, all of which have $\rho >0.5$, while the correlations of adjacent DMR CpGs in dmrff vary in a wide range. In addition, we checked the overlapping DMR CpGs of both methods, and found gbdmr covered most of dmrff’s highly-correlated DMR CpGs. For instance, in Analysis 2, dmrff identified 91 regions with a block size equal to 2, and among them, 60 DMRs were highly correlated ($\rho >0.5$), resulting in 120 CpGs. Gbdmr identified 104 of the 120 DMR CpGs identified by dmrff.

Furthermore, the DMRs identified in this study can be associated with biological and phenotype-related information using the EWAS Open Platform. This platform is a valuable resource that integrates knowledge from existing epigenome-wide association studies (EWAS) [29]. For instance, in Analysis 1, we were able to link the sex-related phenotype to 480 out of 900 DMRs identified by dmrff and 514 out of 1114 DMRs identified by gbdmr. This integration enables a deeper understanding of the potential biological significance of these DMRs in relation to specific traits or conditions.

**Table 1:** Number of DMRs and DMPs identified by different approaches

	dmrff		gbdmr ^1^		EWAS
Analysis index	DMPs	DMRs	DMPs	DMRs	DMPs
1. IOW male vs. female^2^	2085	900 (2418)	1884	1114 (3727)	3435
2. IOW male vs. female^3^	468	148 (391)	423	649 (2352)	697
3. IOW male vs. female^4^	1050	418 (1163)	1092	457 (1494)	1664
4. IOW male vs. female^5^	4226	1986 (5314)	4300	1814 (5798)	7800
5. IOW gestational age^5^	908	333 (907)	996	352 (1120)	1360
6. IOW birth weight^5^	210	78 (220)	219	138 (547)	303
7. GSE59065 male vs. female^6^	946	345 (1008)	1010	448 (2224)	1304
8. GSE59065 young vs. old^6^	20453	12376 (37651)	22814	9252 (27757)	42126
9. GSE87571 male vs. female^7^	3381	1861 (5394)	3575	1613 (5611)	6710
10. GSE87571 age^7^	72070	28586 (87185)	101188	11741 (31409)	133507

Using data in the IOW cohort and GSE datasets. DMPs: differential methylated CpG sites; DMRs: differential methylated regions that contain more than one CpG site. The number of DMR CpGs is included in the parentheses

^1^ The adjacent correlation threshold used for gbdmr is 0.5. ^2^ IOW dataset age 26. ^3^ IOW dataset age 18. ^4^ IOW dataset age 10. ^5^ IOW dataset age at birth. Gestational age is used to describe how far along the pregnancy is. It is measured in weeks

^6^ Age-related profiling of DNA methylation in CD8+ T cells and age is recorded as a binary variable

^7^ Continuous Aging of the Human DNA Methylome Throughout the Human Lifespan Dataset

### Consistency of the findings

To ensure the robustness of our novel approach, we employed a dual strategy for validating its findings. Firstly, we examined the agreement of identified CpGs among various methods within the IOW dataset using Venn plots. Secondly, we conducted cross-validation of the positively identified CpGs across diverse datasets and for identical phenotypes.

**Fig. 4:**
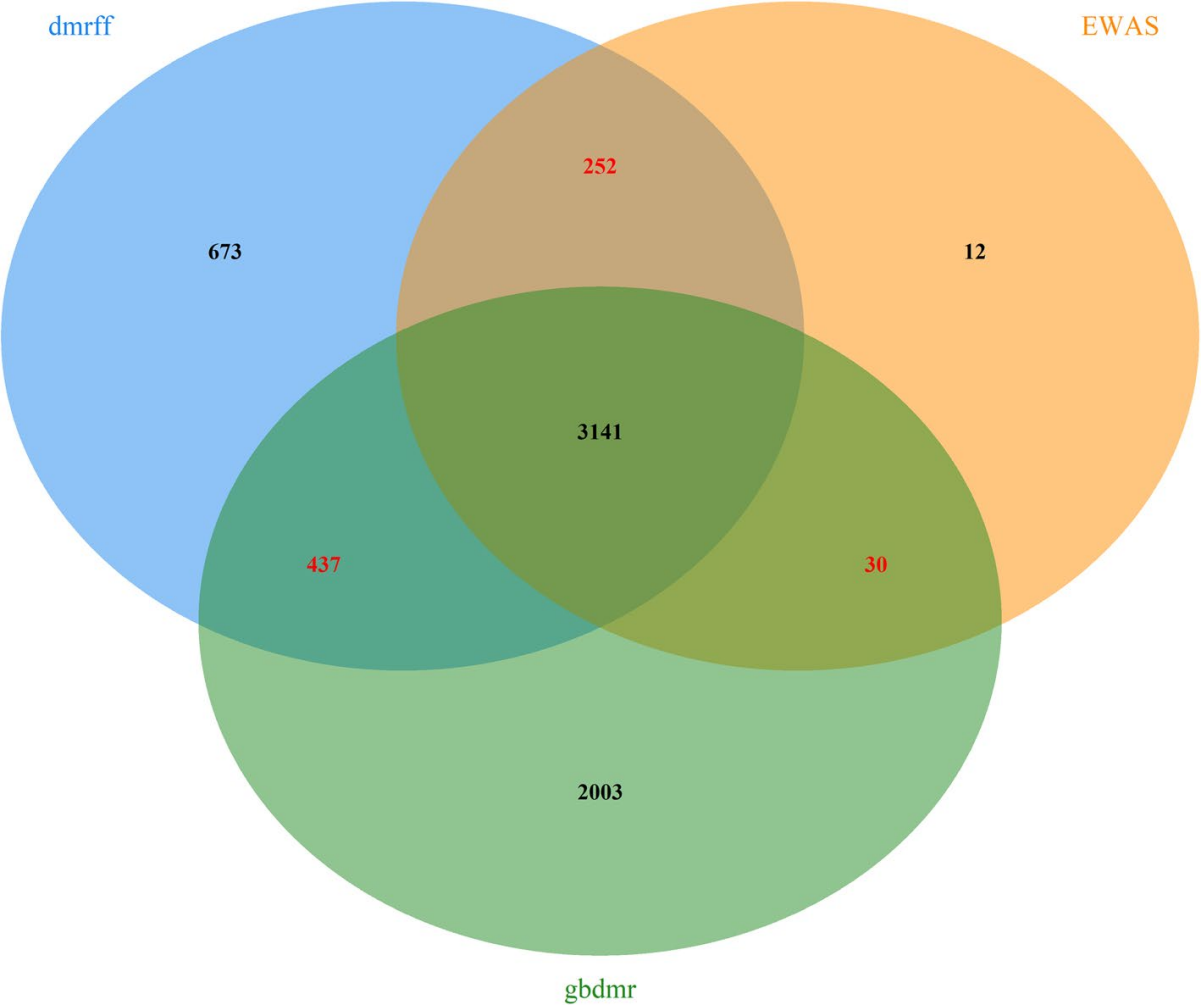
Venn plot for identified CpGs across different approaches

Figure 4 illustrates a Venn plot displaying sex-associated CpGs in Analysis 1 (Table 1). Remarkably, 3141 CpGs, including DMR CpGs (CpGs contained in DMRs) and DMP CpGs (single CpGs) are identified by all three methods. When using EWAS as the benchmark, 98.7% of CpGs from EWAS are identified by dmrff, while the overlapping between EWAS and gbdmr is 92.3%. The higher overlapping rate between EWAS and dmrff can be attributed to dmrff’s reliance on EWAS summary results for subsequent analyses. In Additional file 1: Appendix C, DMPs identified by dmrff and gbdmr show strong consistency. Thus, the primary distinction between dmrff and gbdmr lies in their ability to identify DMRs. Further analysis shows gbdmr can identify a greater number of DMR CpGs, and more CpGs can be associated with the phenotype across different datasets.

To examine the consistency of findings across different datasets, we first applied dmrff, gbdmr and EWAS methods to identify CpGs associated with sex in IOW data at age 26 (Analysis 1 in Table 1), then replicated these analyses in GSE59065 and GSE87571 and recorded the number of CpGs identified in the IOW cohort that are also detected in GSE59065 or GSE87571. We used the IOW data at age 26 to keep consistent with the age of adult samples in GSE59065 and GSE87571. The results are presented in Table 2. The gbdmr identified 767 CpGs (DMR plus DMP CpGs) in GSE59065 and 3158 CpGs in GSE87571 that overlap with those identified in IOW at age 26 years. Compared to dmrff, gbdmr identified less CpGs based on data in GSE59065 but more in GSE87571. When we exclude the DMP CpGs and focus only on the overlapping DMR CpGs, gbdmr identified more CpGs than dmrff in both datasets.

**Table 2:** Cross-validation of the identified CpGs in other datasets

	Number of overlapping DMP + DMR CpGs			Number of overlapping DMR CpGs	
Dataset	EWAS	dmrff	gbdmr	dmrff	gbdmr
GSE59065	549	797	767	305	376
GSE87571	2108	2731	3158	1291	1922

Among the CpGs identified at age 26 in IOW (Analysis 1 of Table 1), the numbers of CpGs also identified in GSE59065 and GSE87571 across different approaches

### Biological relevant analysis

For the 2003 CpGs uniquely identified by gbdmr shown in Fig. 4, we examined their biological functions through enrichment analyses. We first mapped the unique CpGs to their corresponding genes, and then conducted Gene Ontology (GO) and Kyoto Encyclopedia of Genes and Genomes (KEGG) enrichment analysis. Finally, our analysis revealed the significance of two pathways: Tissue development (adjusted *p*-value of 0.02) and Neuroactive ligand-receptor interaction (adjusted *p*-value of 0.03). With sex as a phenotypic variable of interest, the pathways based on mapped genes of CpGs uniquely identified using gbdmr underline sex differences and support the validity of these extra CpGs. For example, tissue development is the top most pathway in which the corresponding genes are enriched and sex differences related to tissue development have been suggested across different studies[30–32]; A previous research report highlighted a significant sex-related difference in the Neuroactive ligand-receptor interaction pathway during Maternal Immune Activation[33].

**Fig. 5:**
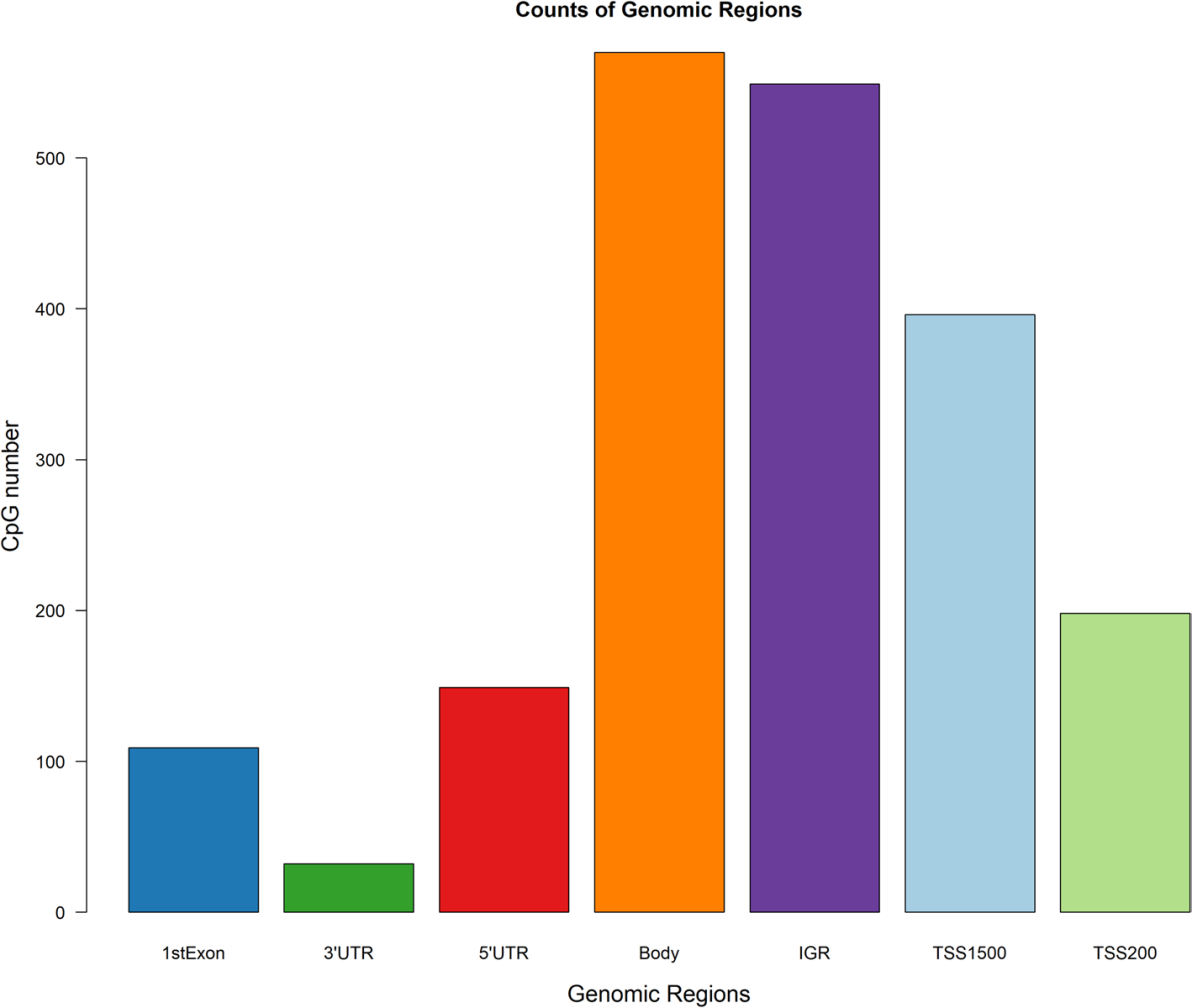
Extra CpGs genomic region distribution

DNAm microarray data typically spans various gene regions, encompassing TSS1500 (200–1500 bases upstream from the transcriptional start site, TSS), TSS200 (0-200 bases upstream from the TSS), 1st Exon, 5’UTR (5’ untranslated region), gene body, 3’UTR (3’ untranslated region), and IGR (intergenic region). We further examined the location of the extra CpGs from gbdmr with respect to genomic regions in Fig. 5 and found that about one-third (29.6%) of the CpGs are in the region of TSS200 and TSS1500. The percentage increased to 42.0% with CpGs at 5’ UTR and 1st Exon also included.

### Program complexity analysis

In Additional file 1: Appendix D, we included the computational complexity for gbdmr, EWAS, and dmrff across different sample sizes and numbers of CpGs. Specifically, Table 1 shows the increase of computing burden as sample size increases with the number of CpGs held constant, while Table 2 displays the changes of computing burden as the number of CpGs increases with sample size held constant. Our findings reveal that gbdmr scales effectively as the sample size and number of CpGs increase, with linear space complexity (*O*(*n*)) and linear time complexity (*O*(*n*)). In the comparison with EWAS and dmrff, gbdmr showed comparable memory-usage while a little bit longer running time. It is important to note that the difference in time efficiency between different methods primarily stems from the optimization processes. Both dmrff and EWAS use least square regression, a well-established technique with highly optimized R implementations. In contrast, gbdmr relies on the Nelder-Mead method, a general-purpose optimization algorithm focusing on numerical maximization of likelihood estimates [34, 35]. We integrated a parallel computing algorithm into gbdmr to ensure its ability to efficiently handle genome-scale DNA methylation data with a reasonably large sample size. Coupled with its linear time complexity and the method’s capability to account for CpG dependence through generalized beta distribution, gbdmr emerges as a valuable tool in the realm of genomics.

### R package implementation

The newly proposed method has been implemented in the R package, gbdmr. The package is available at GitHub: https://github.com/chengzhouwu/gbdmr with a detailed instruction file on how to find the clustered CpG sites, calculate the statistics and extract the CpG information. Gbdmr accommodates both categorical and continuous covariates of interest, and is applicable for various data sources, including 450k BeadChip, 850k BeadChip, or the EPIC array.

## Materials

This section provides the specifics of our simulations and real data analyses.

### Simulation method

To be consistent with the definition of beta values, we used beta distribution to simulate the DNAm levels of single CpG sites. In Figs. 2 and 3, our phenotype is a binary variable representing the presence or absence of a certain phenotype. Given one CpG site, we generated 253/253 DNAm levels, representing samples whose trait is present/absent. We also explored scenarios where the presence/absence of DMRs is unbalanced by generating 422/84 DNAm levels. The results, as reported in Additional file 1: Appendix E, remain consistent with our findings. The total sample size 506 is the same as the Isle of Wight dataset in the real data analysis. To control signal strength, DNAm levels of the two phenotype groups have the same standard deviation and different means by adjusting the shape and rate parameters of beta distributions. The signal strength is defined as the number of standard deviations between the mean of two phenotype groups.

To generate blocks of size $>1$, we first simulated the DNAm levels of a single CpG site following beta distributions. Then, we generated a second vector such that it has a fixed correlation with the first CpG site with the same mean and standard deviation. We followed the same step to generate the third CpG site’s DNAm levels given the second, and so on. This procedure was used to simulate a chain of CpG sites with a given correlation between adjacent CpG sites.

When applying the three methods to the generated datasets, we used the simulated beta values for gbdmr, and used the transformed M values for dmrff and EWAS since these two approaches rely on linear regressions. For the power and false positive assessment in Fig. 2, we generated CpGs’ values under different block sizes = 2 and 3 and different correlation strengths. For Fig. 3, we generated DNAm following the same procedure as in Fig. 2. When the block size is larger than 1, we set the correlation of CpG sites within each block to 0.8. For both simulations, 500 Monte Carlo replicates were generated for the purpose of power estimation and calculations of false positive rate and positive rate.

In addition to binary phenotypes, we extended our investigation to continuous phenotypes. Varied correlations between adjacent CpG sites and signal strengths were systematically employed to assess the performance of the three methods. Detailed results of these simulations, including data generation settings, power, false positive rate, and effect size figures, are in Additional file 1: Appendix F. The simulation results are consistent with the findings for binary phenotypes.

### The three real datasets

We analyzed three DNAm datasets. The first dataset was obtained from a birth cohort study conducted on the Isle of Wight in the United Kingdom. DNAm levels were measured in whole blood at different ages (birth, age 10, age 18, age 26) and preprocessed by background correlation, normalization, and batch effect removal. The final dataset contains 346,009 CpG sites with sample sizes ranging from 277 to 506. The other two datasets were obtained from the Gene Expression Omnibus (GEO) database repository. The first dataset (GSE59065) included 101 individuals (50 young and 51 old) and focused on age-related profiling of DNA methylation in CD8+ T cells. The second dataset (GSE87571) included 732 samples and investigated the continuous aging of the human DNA methylome throughout the lifespan.

For all the analyses, we used Bonferroni correction to adjust *p*-values. For dmrff, we divided regions using the default maximum distance of 500 bp. For gbdmr, we segmented blocks using a correlation threshold of 0.5.

## Discussion

**Fig. 6:**
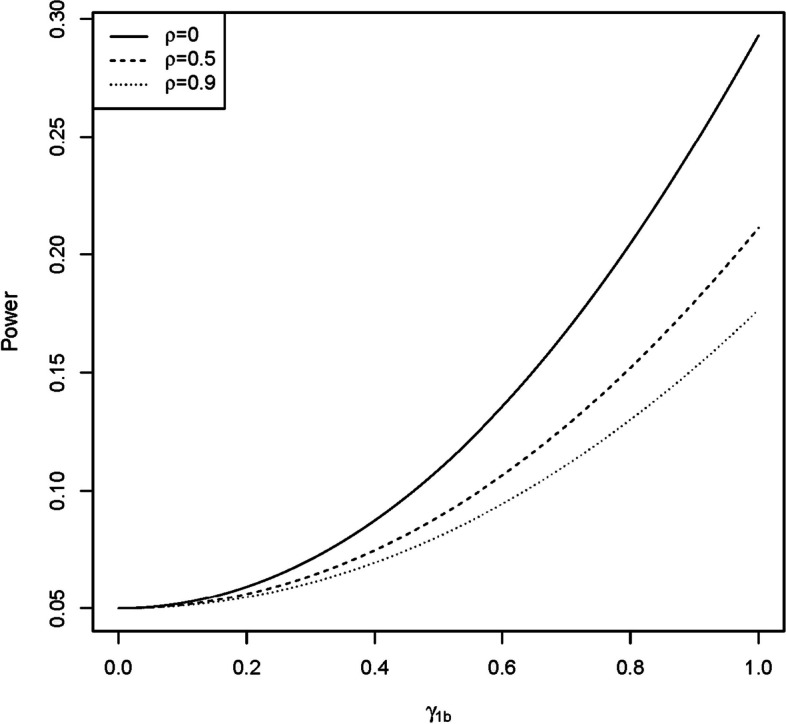
Power of dmrff when true $\gamma _{1b}$ ranges from 0 to 1

Different from the meta-analysis methods that summarize the EWAS results, the proposed method, gbdmr, is a model-based approach that fits the DNAm data by generalized beta distribution. In the simulation study, we demonstrated that the dmrff was less efficient when the correlation between adjacent CpG sites was strong. This is counter-intuitive since DMR detection methods are expected to achieve a better performance in strong correlations. In Additional file 1: Appendix G, We show that the power of dmrff equals

$$\begin{aligned} \text{ Power }=P\left( Z>-\frac{\gamma _{1b}}{\sqrt{(\pmb {1}^\top \pmb {\Omega }^{-1}\pmb {1})^{-1}}}+z_{1-\alpha /2}\right) \\ +P\left( Z<-\frac{\gamma _{1b}}{\sqrt{(\pmb {1}^\top \pmb {\Omega }^{-1}\pmb {1})^{-1}}}+z_{\alpha /2}\right) , \end{aligned}$$

where $\gamma _{1b}$ is the true effect of phenotype in Eq. (2); $\pmb {1}=(1,\dots ,1)^\top$;

$$\begin{aligned} \pmb {\Omega }=\sigma _n^2 \begin{bmatrix} 1 &{} \rho &{} \ldots &{} \rho \\ \rho &{} 1 &{} \ldots &{} \rho \\ \vdots &{} \vdots &{} \ddots &{} \rho \\ \rho &{} \rho &{} \rho &{} 1 \end{bmatrix}, \end{aligned}$$

an $L_b$ by $L_b$ matrix where $L_b$ is the number of CpG sites in *b*th DMR; $\sigma _n=\sigma /\sqrt{\sum _{i=1}^n(X_i-{\bar{X}})^2}$; $\sigma$ is the standard deviation of the DNAm levels of a CpG site; $X_i$ is the phenotype of the *i*th sample; ${\bar{X}}=\sum _{i=1}^n X_i /n$; $\rho$ is the pairwise correlation among CpG sites in a DMR; *Z* is a random variable following standard normal distribution; $z_{1-\alpha /2}$ and $z_{\alpha /2}$ are $1-\alpha /2$ and $\alpha /2$ quantile of the standard normal. Figure 6 shows the power of dmrff versus the effect size $\gamma _{1b}$ when $\sigma _n=1$, $L_b=2$, $\rho =0$, 0.5, and 0.9.

From Fig. 6, we observe that the theoretical power of dmrff declines as $\rho$ increases. Here we provide an intuitive explanation of the theoretical results. The dmrff summarizes *p* single CpG sites’ effects $\pmb {{\widehat{\beta }}}=(\widehat{\beta }_1,\dots , {\widehat{\beta }}_p)^\top$ in the same region, where $\pmb {{\widehat{\beta }}}$ is the EWAS effect estimates of *p* CpG sites. Under a simplified condition that $\sigma$ is known, dmrff is equivalent to dividing the weighted average $A=(\pmb {1}^\top \pmb {\Omega }^{-1}\pmb {1})^{-1}\pmb {1}^\top \pmb {\Omega }^{-1} \pmb {{\widehat{\beta }}}$ by its standard error $\sqrt{(\pmb {1}^\top \pmb {\Omega }^{-1}\pmb {1})^{-1}}$ as the test statistics. We examine two extreme cases: If the *p* CpG sites are mutually independent, then $\rho =0$ and $\pmb {\Omega }$ becomes a diagonal matrix. The dmrff is equivalent to a one-sample Z-test of sample size *p* with i.i.d observations ${\widehat{\beta }}_1, \dots , {\widehat{\beta }}_p$. On the other side, if *p* CpGs are perfectly correlated, i.e., $\rho =1$, ${\widehat{\beta }}_1$ to ${\widehat{\beta }}_p$ will be exactly the same. In this case, there is only one effective observation, and the equivalent sample size is only one. This trend indicates that dmrff has a lower efficiency when the correlation grows stronger. Moreover, this explanation is not only restricted to dmrff, but may apply to a family of methods based on meta-analysis: Given a fixed number of CpG sites, a stronger inter-correlation means higher proportion of overlapping information among CpG sites, and thus fewer equivalent sample sizes can be used in summarizing the results. In contrast, gbdmr uses generalized beta distribution to directly model all CpG sites in a region and is not affected by the equivalent sample size shrinkage in meta-analysis approaches.

## Conclusion

We proposed a novel model-based method for detecting DMRs, called gbdmr, which employs generalized beta regression to model correlated CpG sites. The package gbdmr, unlike traditional methods, does not necessitate the normality assumption on DNA methylation (DNAm) levels and is adept at considering the correlation structures among CpG sites. This approach exhibits a strong ability to identify informative CpG regions, especially in scenarios where there is a high degree of inter-correlation among these sites. The simulation studies show that gbdmr performed better than dmrff and traditional EWAS when the correlation between adjacent CpGs is high, while the dmrff achieves higher power when the correlation is weak. Both theoretical and heuristic explanations are provided for the performance decay of dmrff as correlation increases. Based on the real data analysis, gbdmr was able to identify a higher number of DMR CpGs compared to dmrff in most of the analyses. Further examination revealed that gbdmr was able to identify most of dmrff’s DMRs that exhibit high correlations between adjacent CpGs. These findings are consistent with the results obtained from the simulation study. In the future, a promising approach would be to combine the strengths of dmrff and gbdmr to better adapt to different situations.

### Supplementary Information

**Additional file 1. Supplementary Information** - gbdmr: Identifying differentially methylated CpG regions in the human genome via generalized beta regressions.

## Data Availability

The raw data for GSE59065 and GSE87571 are available at: https://www.ncbi.nlm.nih.gov/geo/. Gbdmr has been implemented in the R software and is available through GitHub: https://github.com/chengzhouwu/gbdmr.
