# Supplementary material for: Gbdmr: identifying differentially methylated CpG regions in the human genome via generalized beta regressions
Source: BMC Bioinformatics. 2024 Mar 5;25:97. doi: 10.1186/s12859-024-05711-y (PMC10916021; doi:10.1186/s12859-024-05711-y)
Supplement: Supplementary file 1 — Additional file 1. Supplementary Information - gbdmr: Identifying differentially methylated CpG regions in the human genome via generalized beta regressions. [file 12859_2024_5711_MOESM1_ESM.pdf]

Supplementary Information - gbdmr: Identifying differentially methylated CpG regions in the human genome via generalized beta regressions

Chengzhou Wu[1], Xichen Mou[1*], Hongmei Zhang[1]

[1*]School of Public Health, University of Memphis, 3720 Alumni Ave, Memphis, 38152, TN, USA.

*Corresponding author(s). E-mail(s): xmou@memphis.edu;
Contributing authors: cwu8@memphis.edu; hzhang6@memphis.edu;

Keywords: Differentially methylated regions, Generalized beta distribution, DNA methylation, CpG site

The supplementary information includes the outcomes of additional simulations, real data analysis, computational complexity, and a comprehensive theoretical power analysis of the dmrff method.

Appendix A

To comprehensively compare different methods' power and false positive rate across different block sizes, we systematically explored various settings, encompassing adjustments in block size, mean values, and standard deviations for each group.

Appendix A.1

To enhance the realism of our simulation, we present the distribution of block sizes within the real data (IOW, GSE59065, GSE87571) for both gbdmr and dmrff. In gbdmr, blocks are determined based on correlation. A chain of neighboring CpGs forms a block if the correlation of each neighboring pair is larger than the correlation threshold. In the histogram, we set the threshold to be 0.5. In dmrff, a block is formed based on the distance. If the distance between two neighboring CpGs is less than 500 bp, then they belong to the same block. As illustrated in Appendix A.1, the majority

of candidate block sizes are clustered within the range of one to ten, with larger regions being infrequent and characterized by very low frequencies. Specifically, we found more than 99.9% of block sizes range between 1 and 10, and over 99.8% of block sizes are less than or equal to 6 across the three datasets.

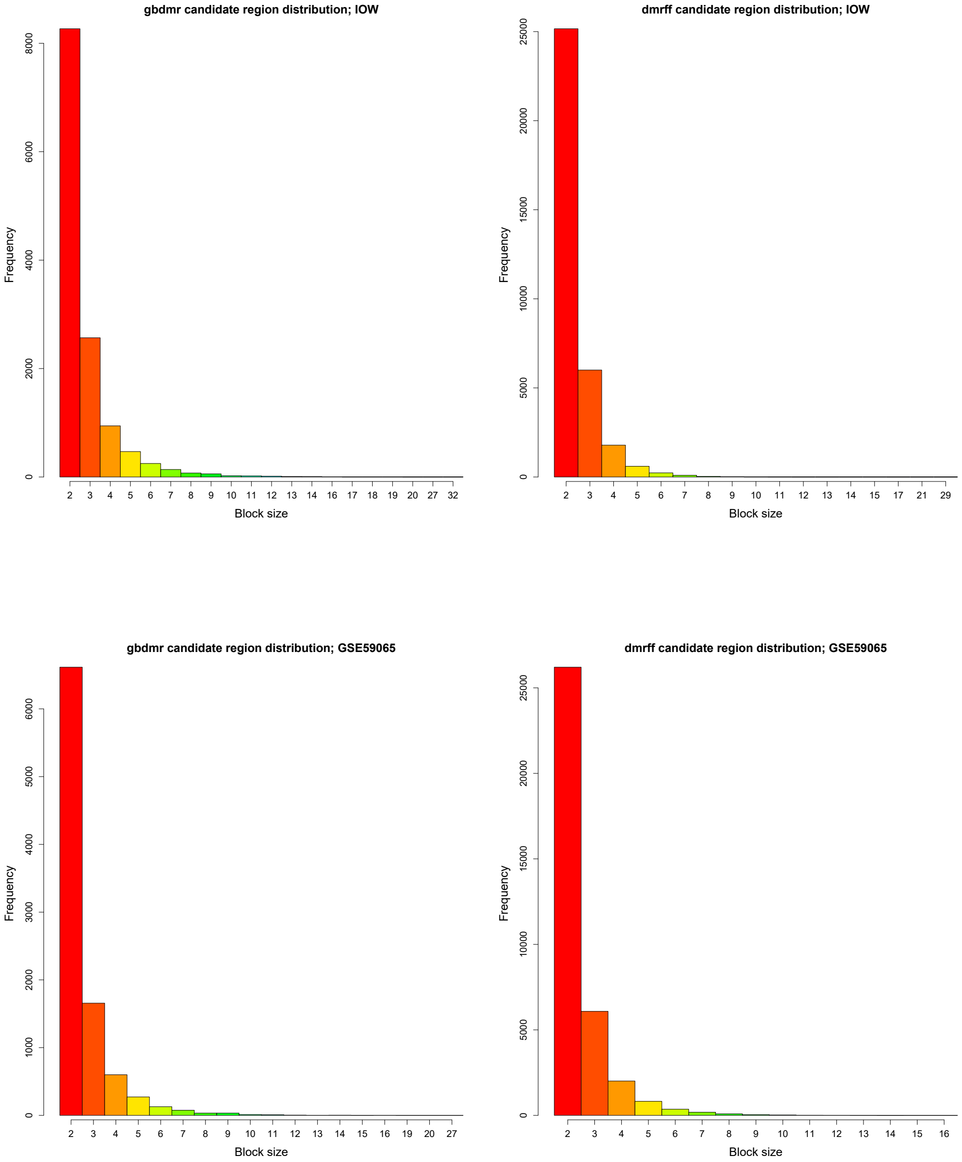

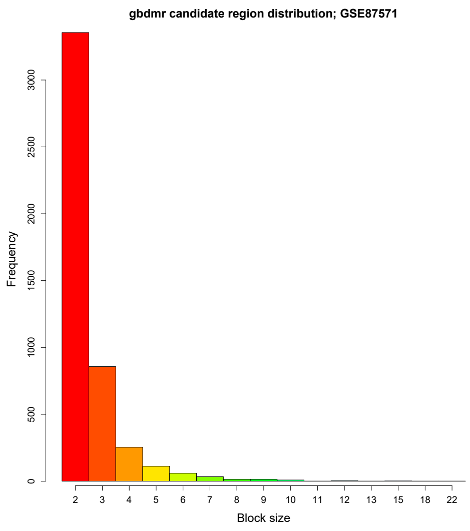
gbdmr candidate region distribution; GSE87571
Frequency
0
500
1000
1500
2000
2500
3000
2 3 4 5 6 7 8 9 10 11 12 13 15 18 22
Block size

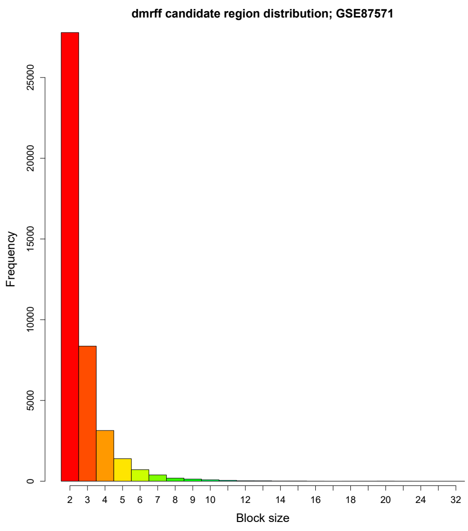
dmrff candidate region distribution; GSE87571
Frequency
0
5000
10000
15000
20000
25000
2 3 4 5 6 7 8 9 10 12 14 16 18 20 24 32
Block size

Appendix A.2

In this appendix, we assess the power and false positive rate of gbdmr, dmrff, and EWAS across additional settings. Specifically, we simulated 253/253 DNAm to represent the trait present/absent groups following gamma distribution. The average DNAm of the two groups are set to be M1 and M2. We examined a spectrum of M1 & M2 and correlation thresholds for ρ. All simulations are repeated 500 times, and the average power/false positive rates are presented in the figures.

When the average DNAm is 0.3:

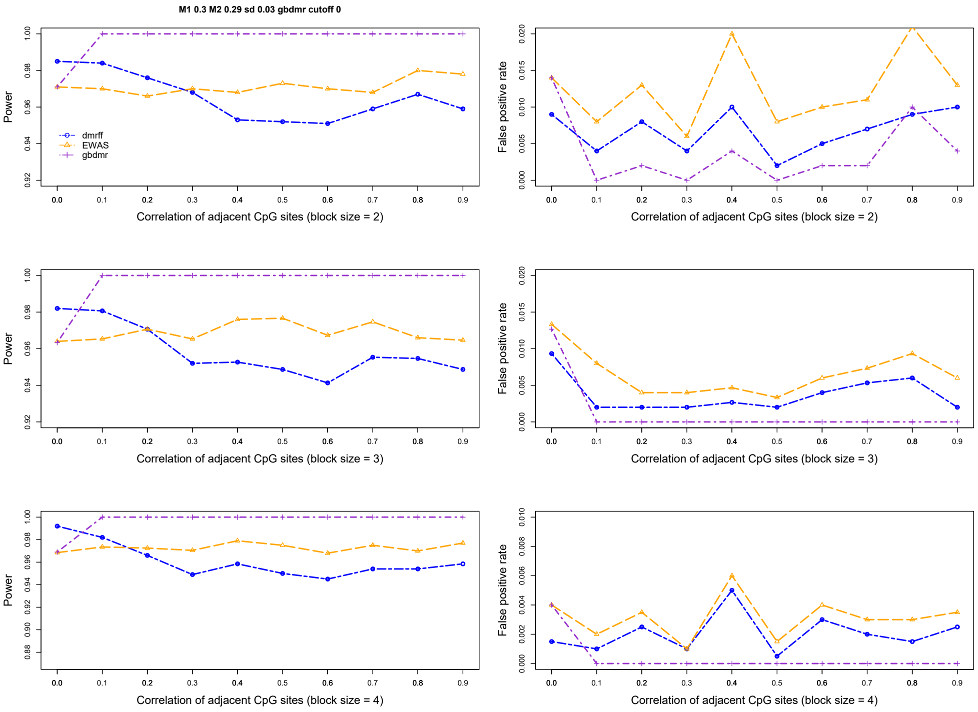

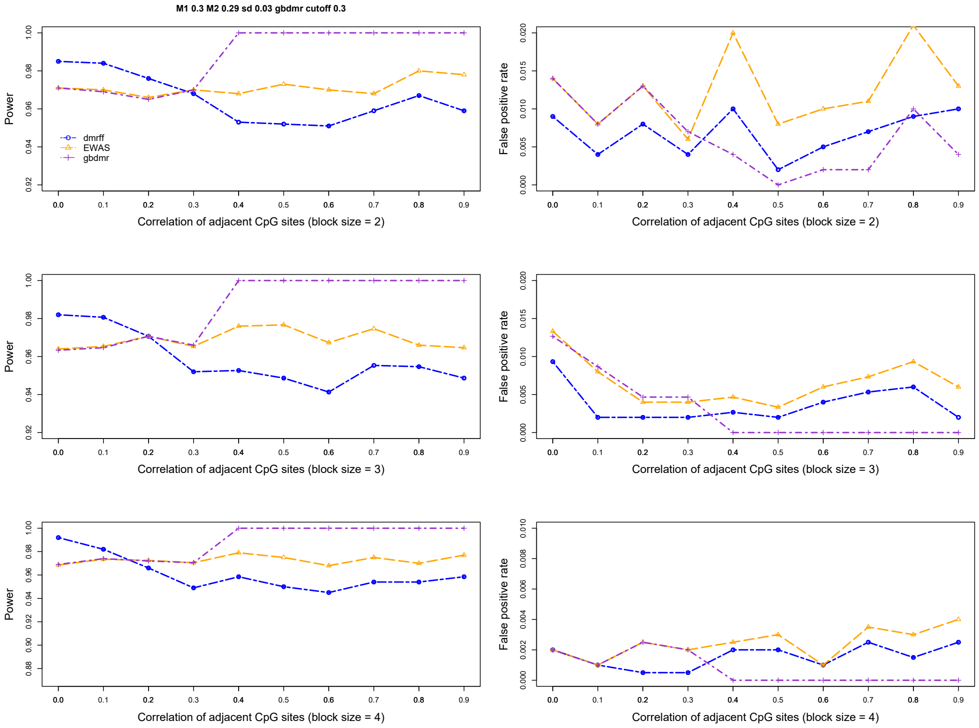
M1 0.3 M2 0.29 sd 0.03 gbdmr cutoff 0.3
dmrff
EWAS
gbdmr
Power
False positive rate
Correlation of adjacent CpG sites (block size = 2)
Correlation of adjacent CpG sites (block size = 3)
Correlation of adjacent CpG sites (block size = 4)

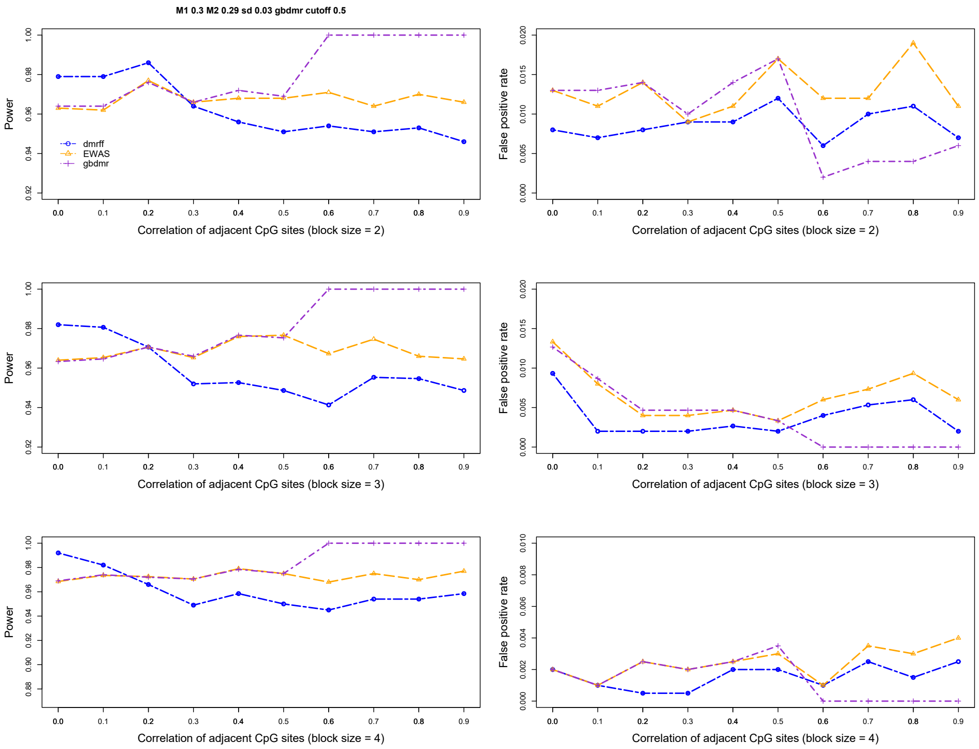
M1 0.3 M2 0.29 sd 0.03 gbdmr cutoff 0.5
Power
dmrff
EWAS
gbdmr
Correlation of adjacent CpG sites (block size = 2)
False positive rate
Correlation of adjacent CpG sites (block size = 2)
Power
Correlation of adjacent CpG sites (block size = 3)
False positive rate
Correlation of adjacent CpG sites (block size = 3)
Power
Correlation of adjacent CpG sites (block size = 4)
False positive rate
Correlation of adjacent CpG sites (block size = 4)

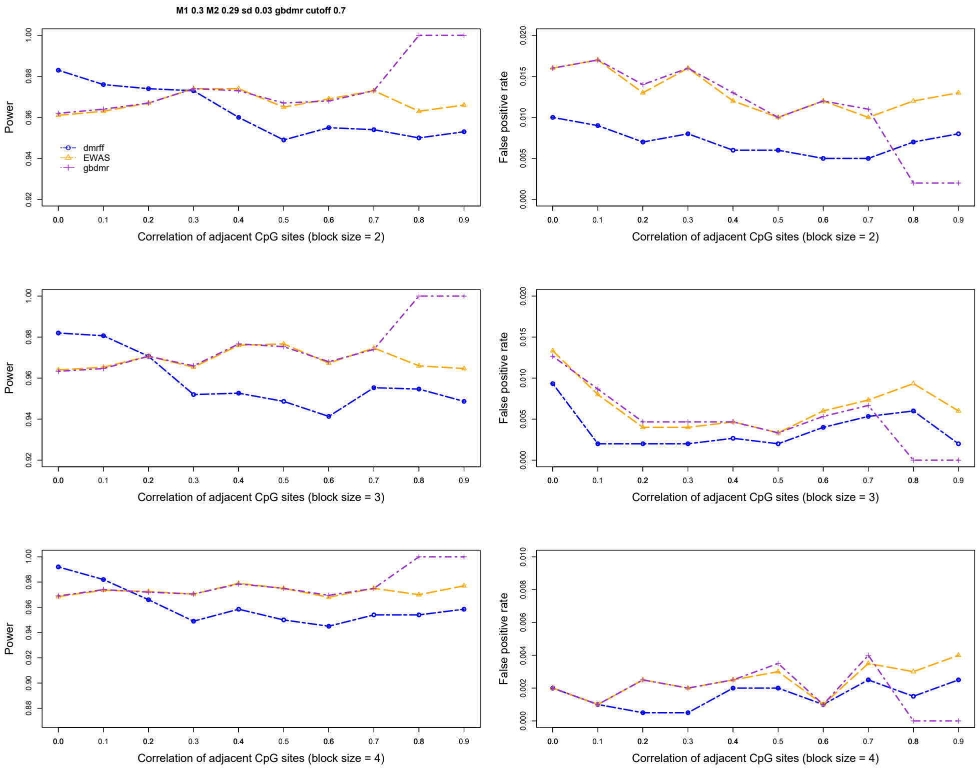
M1 0.3 M2 0.29 sd 0.03 gbdmr cutoff 0.7
Power
False positive rate
dmrff
EWAS
gbdmr
Correlation of adjacent CpG sites (block size = 2)
Correlation of adjacent CpG sites (block size = 3)
Correlation of adjacent CpG sites (block size = 4)

When the average DNAm is 0.5:

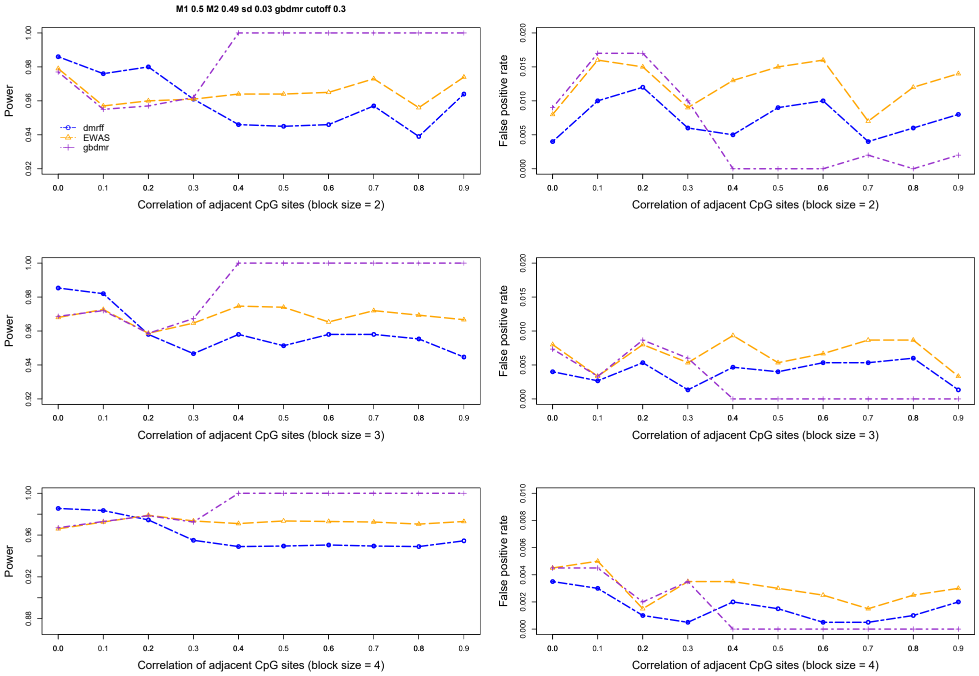

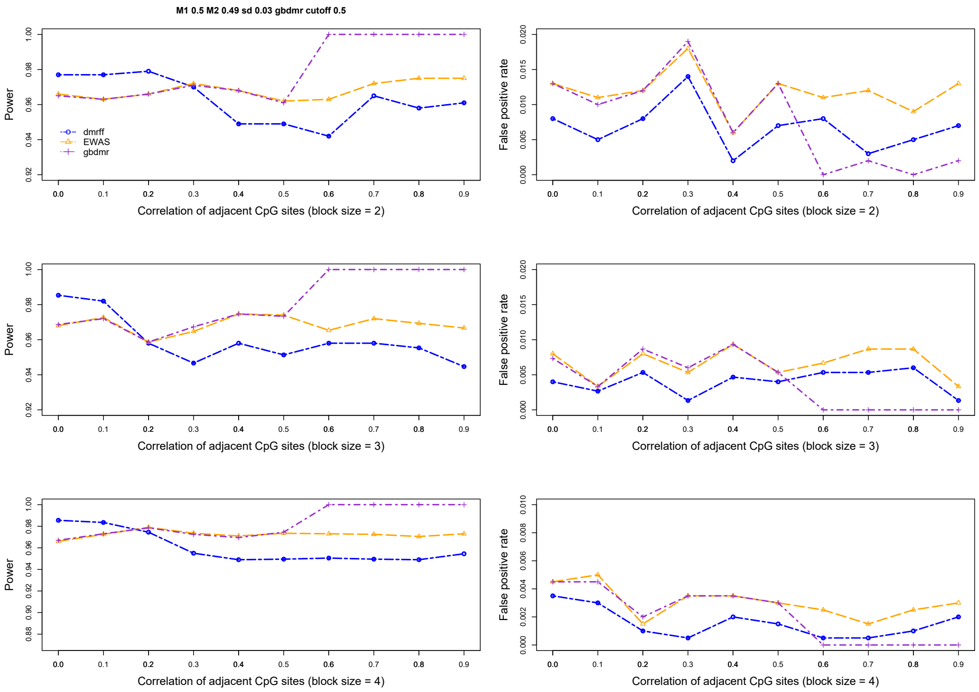
M1 0.5 M2 0.49 sd 0.03 gbdmr cutoff 0.5
dmrff
EWAS
gbdmr
Power
False positive rate
Correlation of adjacent CpG sites (block size = 2)
Correlation of adjacent CpG sites (block size = 3)
Correlation of adjacent CpG sites (block size = 4)

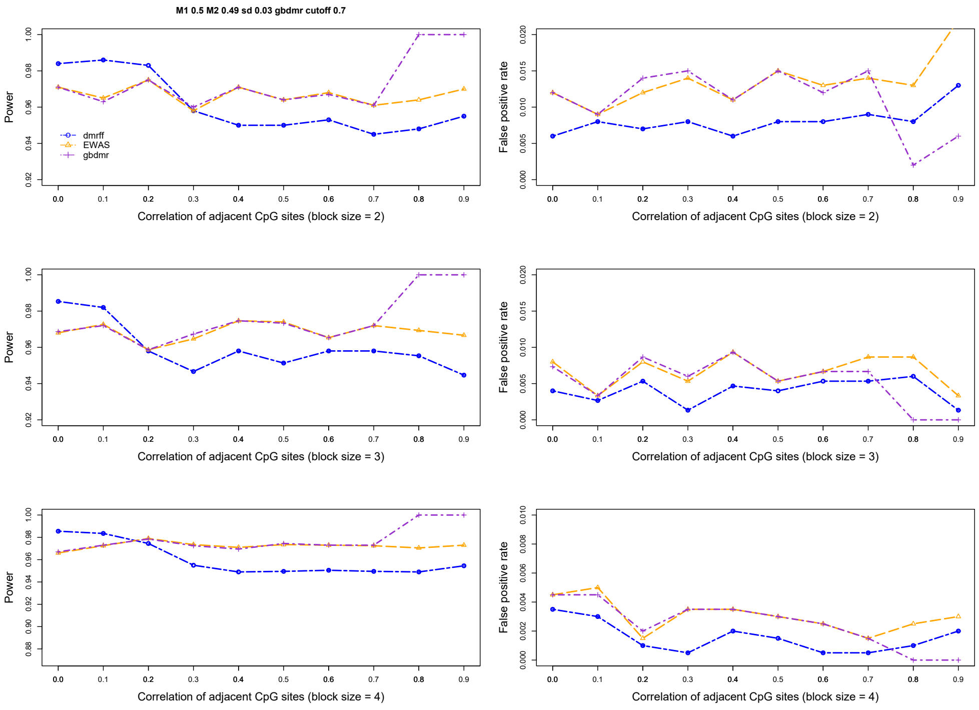
M1 0.5 M2 0.49 sd 0.03 gbdmr cutoff 0.7
dmrff
EWAS
gbdmr
Power
False positive rate
Correlation of adjacent CpG sites (block size = 2)
Correlation of adjacent CpG sites (block size = 3)
Correlation of adjacent CpG sites (block size = 4)

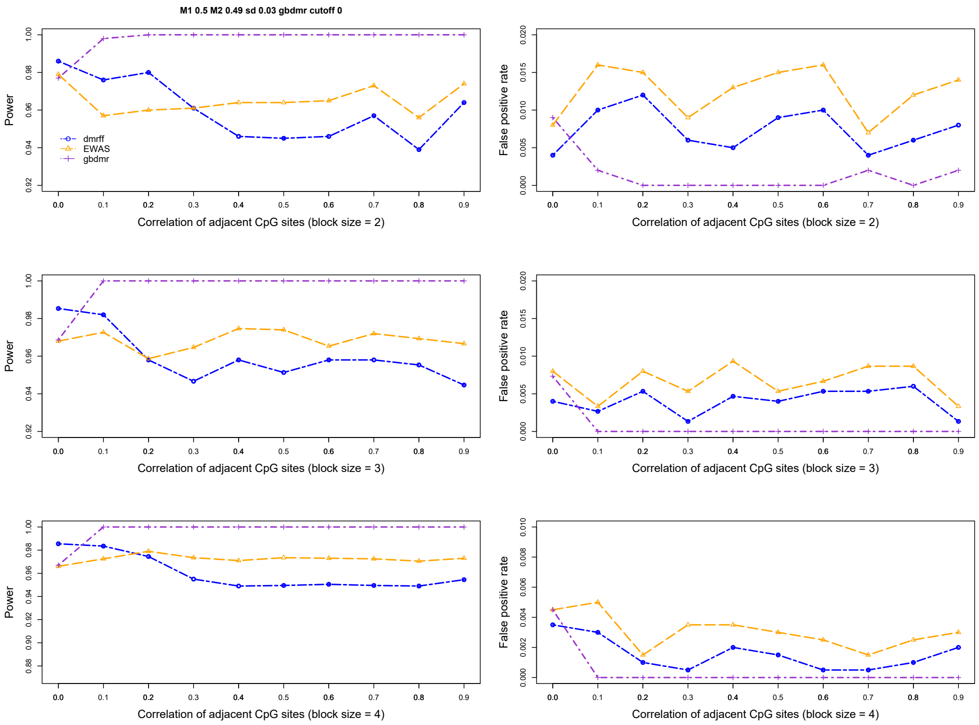
M1 0.5 M2 0.49 sd 0.03 gbdmr cutoff 0
dmrff
EWAS
gbdmr
Power
False positive rate
Correlation of adjacent CpG sites (block size = 2)
Correlation of adjacent CpG sites (block size = 3)
Correlation of adjacent CpG sites (block size = 4)

When the average DNAm is 0.7:

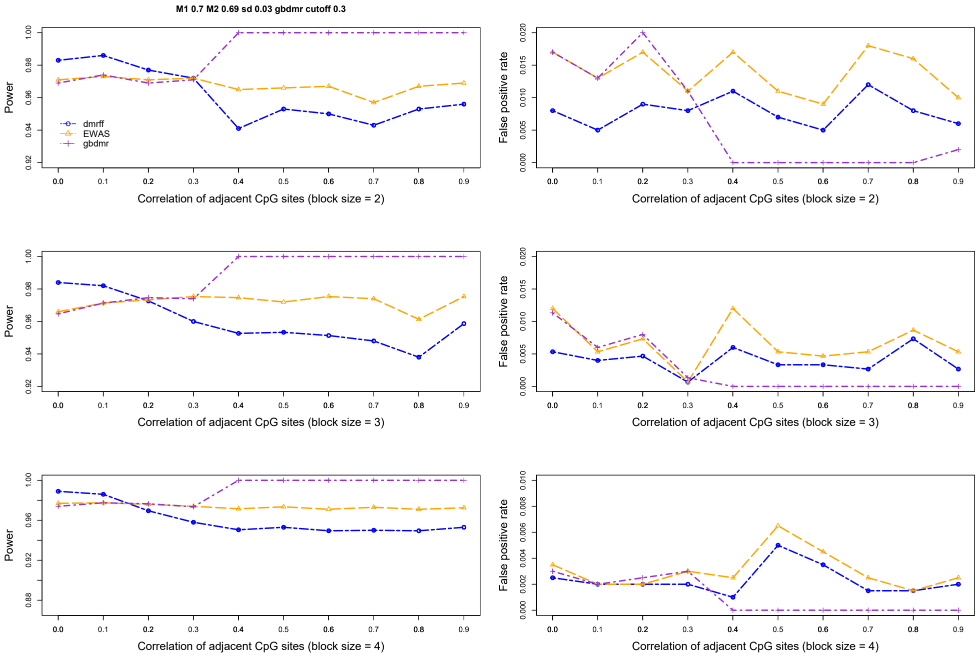

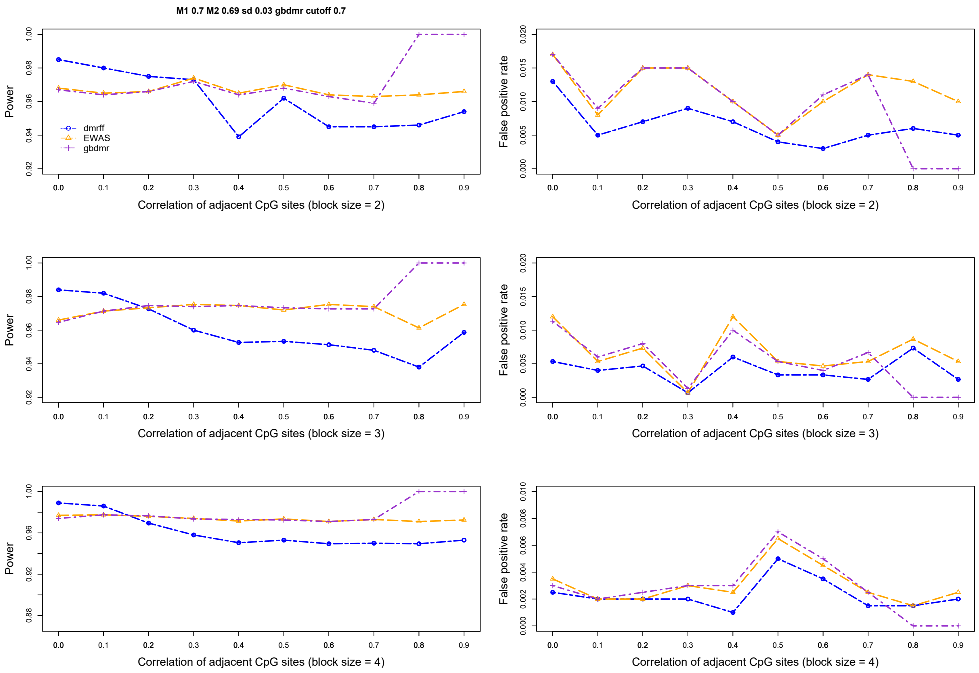
M1 0.7 M2 0.69 sd 0.03 gbdmr cutoff 0.7
Power
False positive rate
dmrff
EWAS
gbdmr
Correlation of adjacent CpG sites (block size = 2)
Correlation of adjacent CpG sites (block size = 3)
Correlation of adjacent CpG sites (block size = 4)

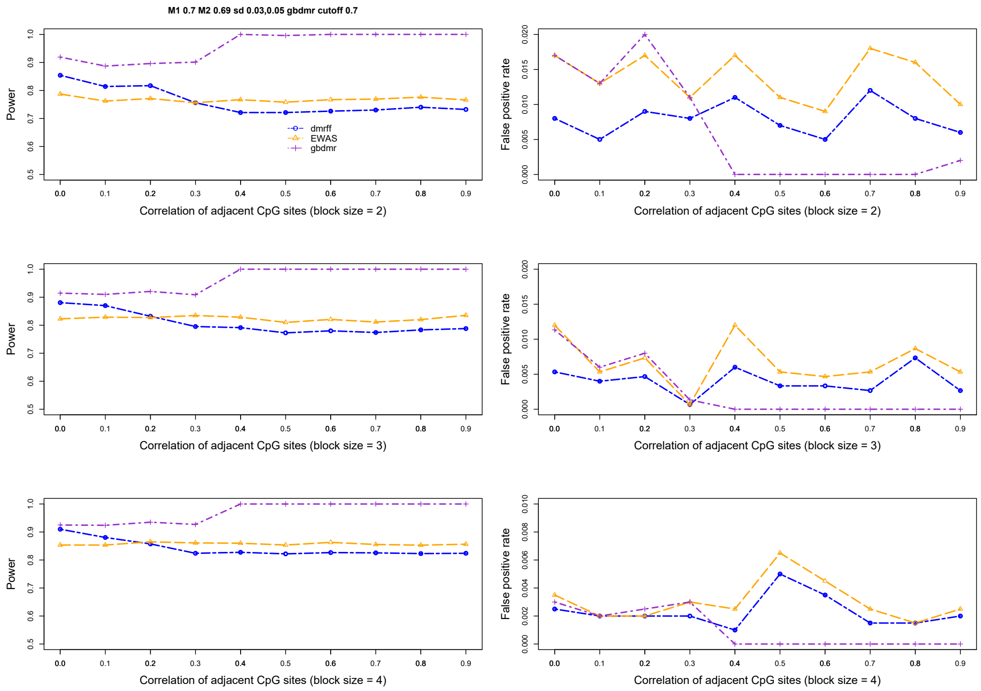
M1 0.7 M2 0.69 sd 0.03,0.05 gbdmr cutoff 0.7
dmrff
EWAS
gbdmr
Power
False positive rate
Correlation of adjacent CpG sites (block size = 2)
Correlation of adjacent CpG sites (block size = 3)
Correlation of adjacent CpG sites (block size = 4)

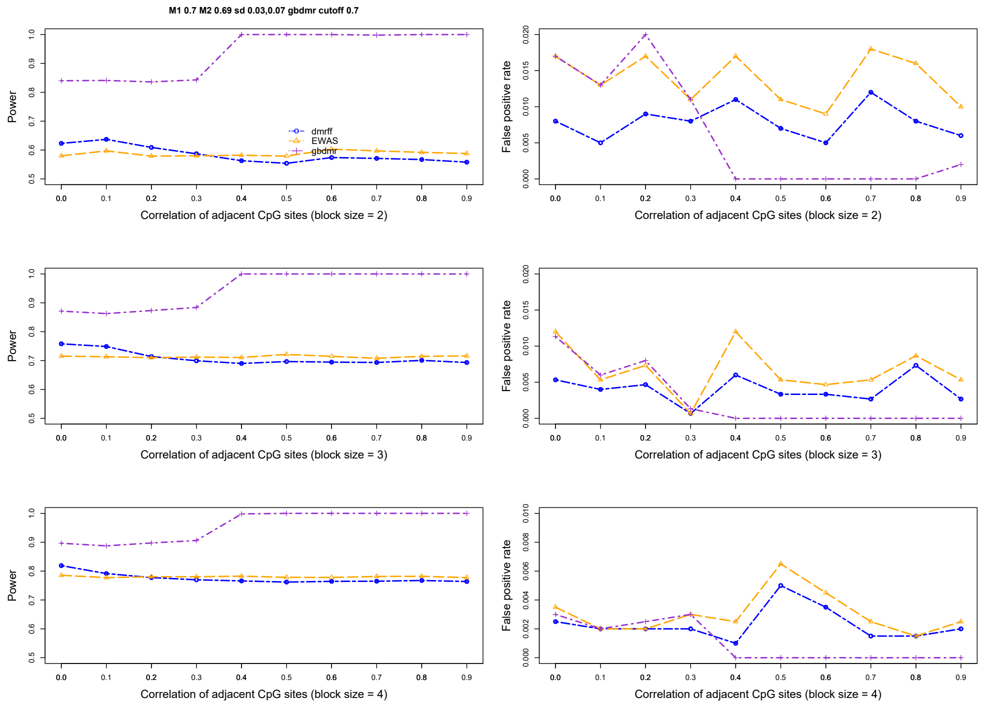
M1 0.7 M2 0.69 sd 0.03,0.07 gbdmr cutoff 0.7
Power
False positive rate
dmrff
EWAS
gbdmr
Correlation of adjacent CpG sites (block size = 2)
Correlation of adjacent CpG sites (block size = 3)
Correlation of adjacent CpG sites (block size = 4)

Appendix A.3

In addition to simulations in A.2, we expanded our analysis to incorporate larger block sizes ranging from 5 to 10. This broader scope allowed us to comprehensively evaluate the system's performance by assessing both power and false positive rates. These evaluations were conducted under average DNAm at 0.3, 0.5, and 0.7. All simulations are repeated 500 times, and the average power/false positive rates are presented in the figures.

When the average DNAm is 0.3:

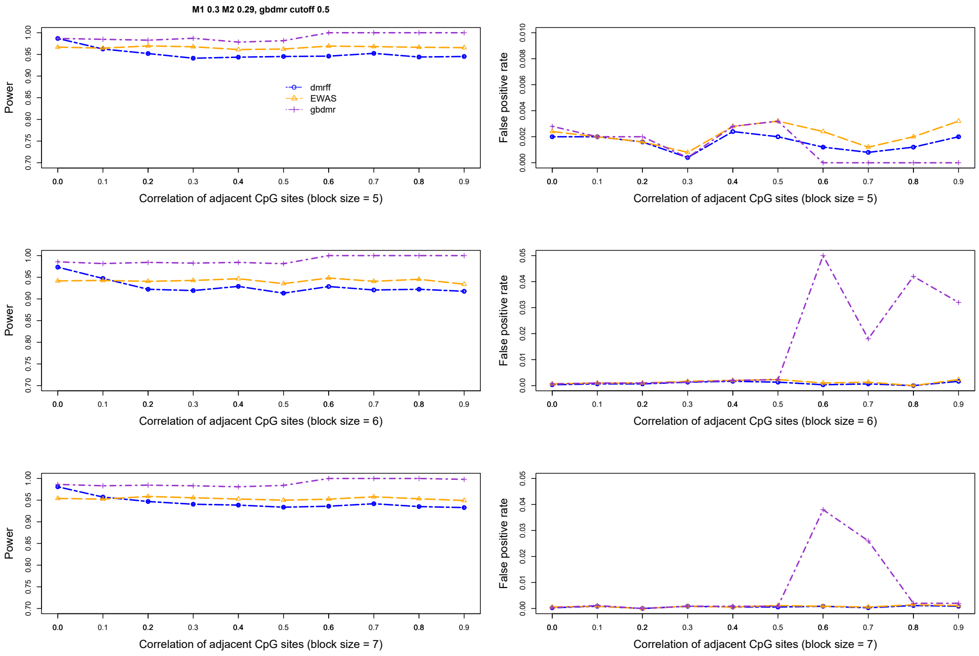

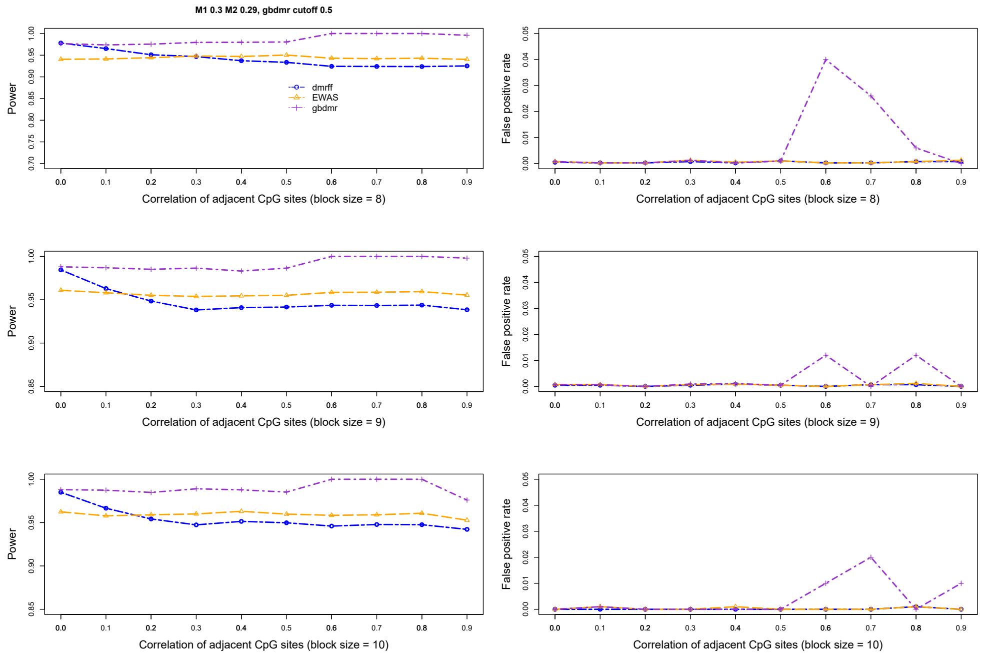

When the average DNAm is 0.5:

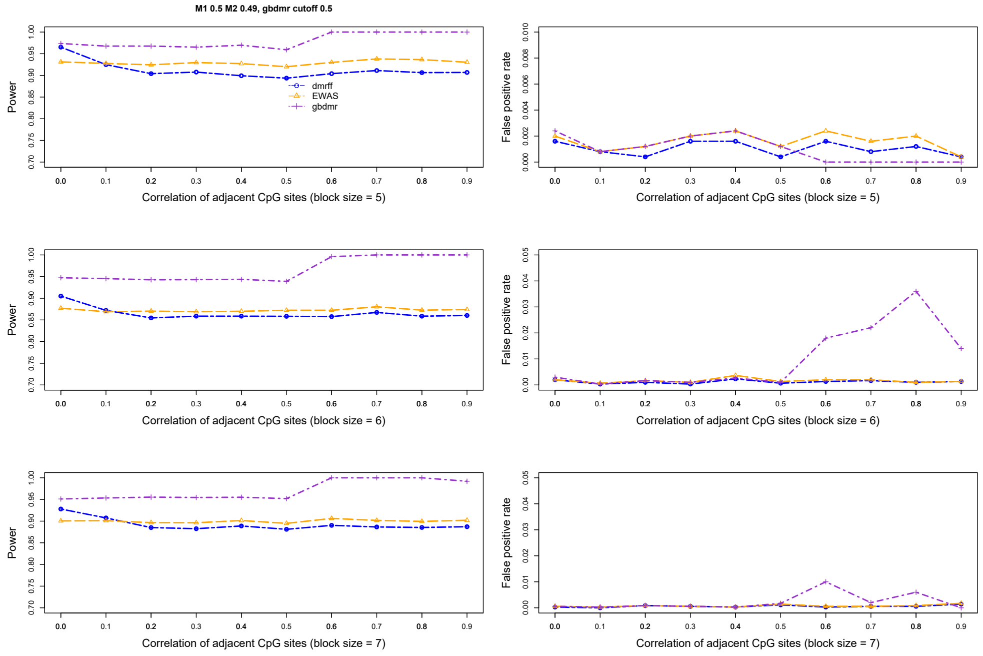

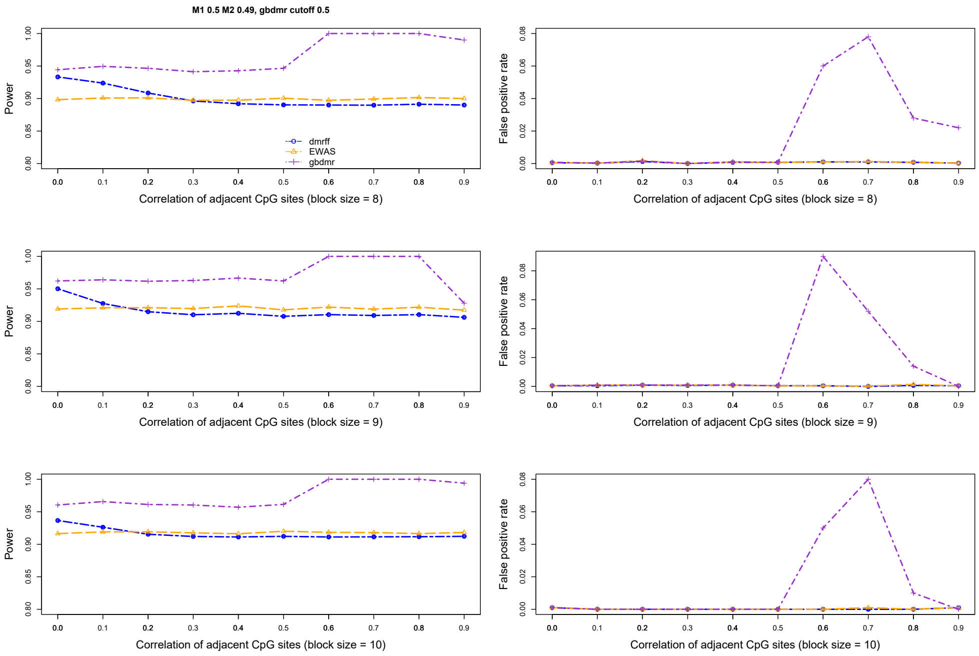
M1 0.5 M2 0.49, gbdmr cutoff 0.5
Power
False positive rate
dmrff
EWAS
gbdmr
Correlation of adjacent CpG sites (block size = 8)
Correlation of adjacent CpG sites (block size = 9)
Correlation of adjacent CpG sites (block size = 10)

When the average DNAm is 0.7:

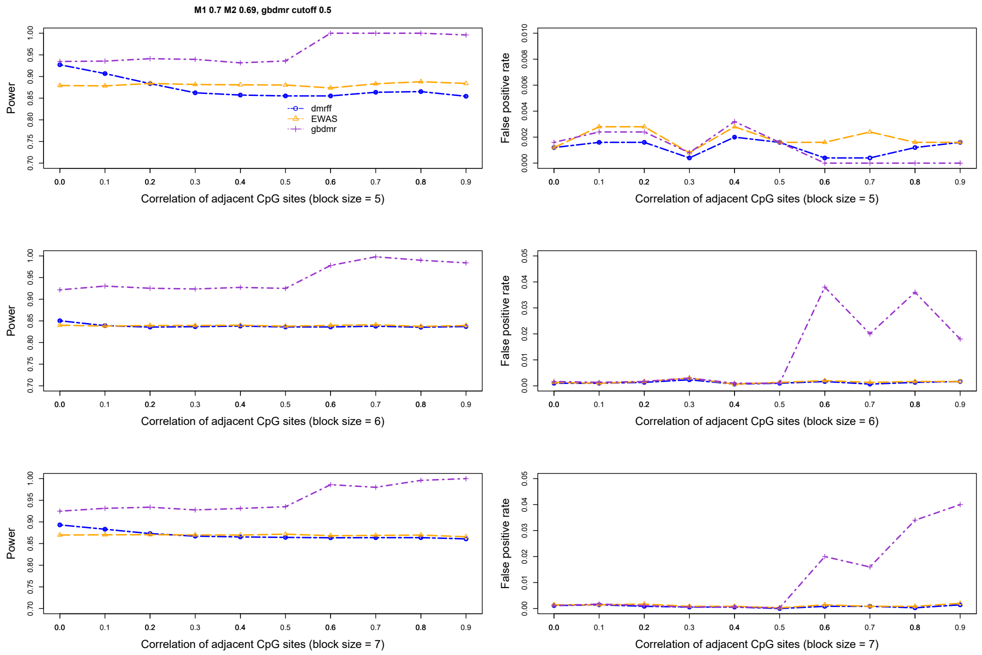

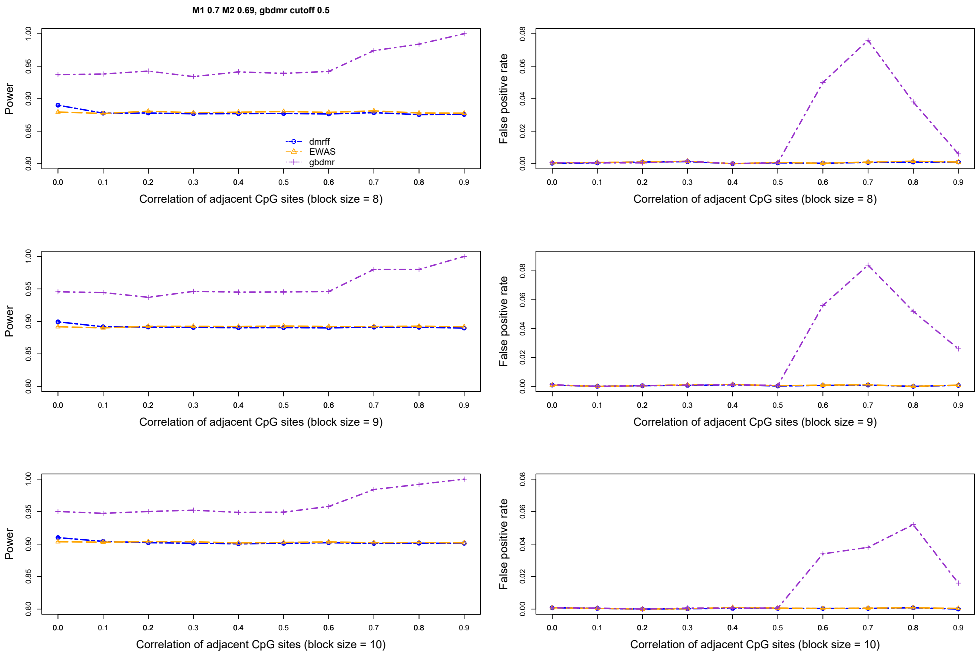
M1 0.7 M2 0.69, gbdmr cutoff 0.5
Power
False positive rate
dmrff
EWAS
gbdmr
Correlation of adjacent CpG sites (block size = 8)
Correlation of adjacent CpG sites (block size = 9)
Correlation of adjacent CpG sites (block size = 10)

Appendix B

In Appendix B, we examined the relationship between signal strength and the power of gbdmr, dmrff, and EWAS. Same as Section A, we simulated 253/253 DNAm to represent the trait present/absent groups following gamma distribution. The signal strength is defined as the mean difference between trait present/absent groups divided by the standard deviation. We checked the performance across different combinations of the block size(1-10), mean, standard deviation of DNAm, gbdmr correlation threshold, and the true correlations between adjacent CpG sites when block size > 1. All simulations are repeated 500 times, and the average power/false positive rates are presented in the figures.

When the average DNAm starts from 0.3:

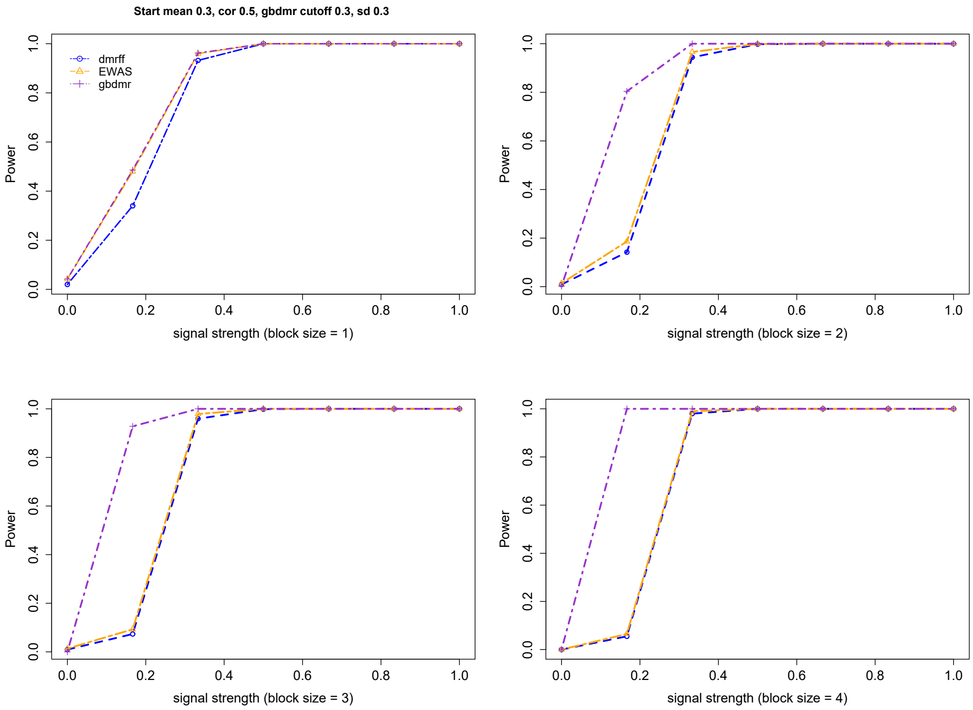

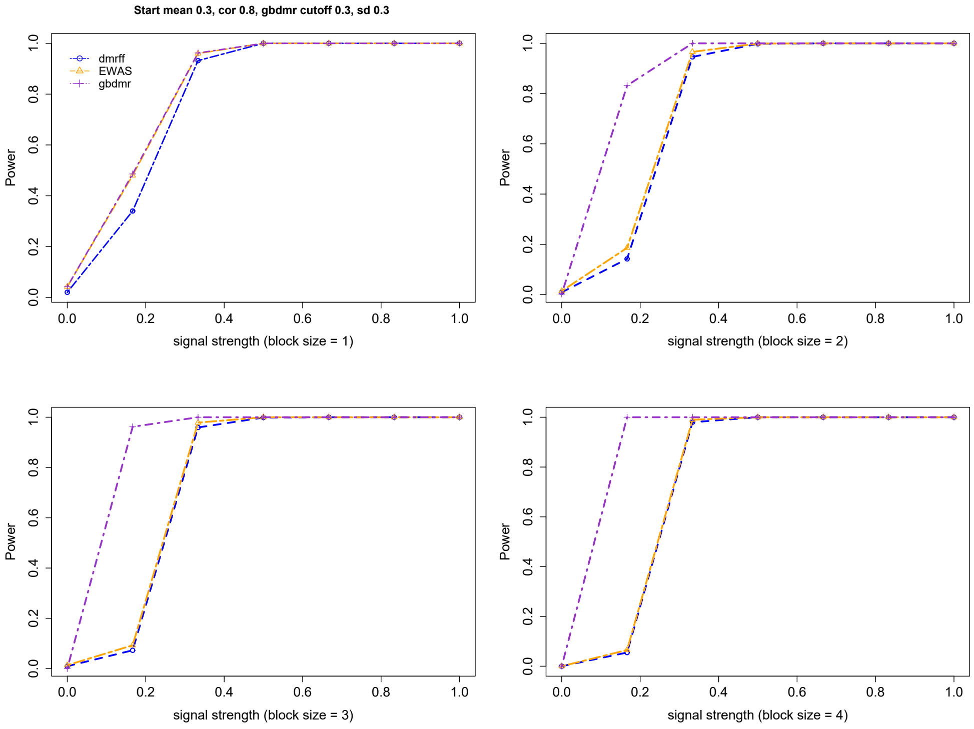

Start mean 0.3, cor 0.8, gbdmr cutoff 0.3, sd 0.3
dmrff
EWAS
gbdmr
Power
signal strength (block size = 1)
signal strength (block size = 2)
signal strength (block size = 3)
signal strength (block size = 4)

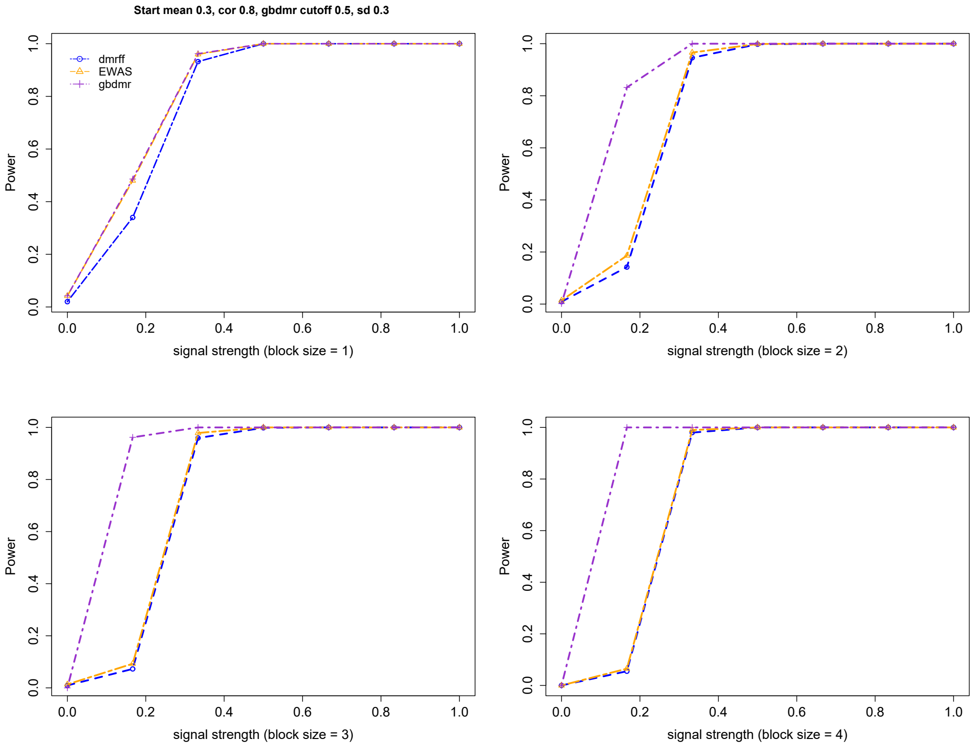

Start mean 0.3, cor 0.8, gbdmr cutoff 0.5, sd 0.3
dmrff
EWAS
gbdmr
Power
signal strength (block size = 1)
signal strength (block size = 2)
signal strength (block size = 3)
signal strength (block size = 4)

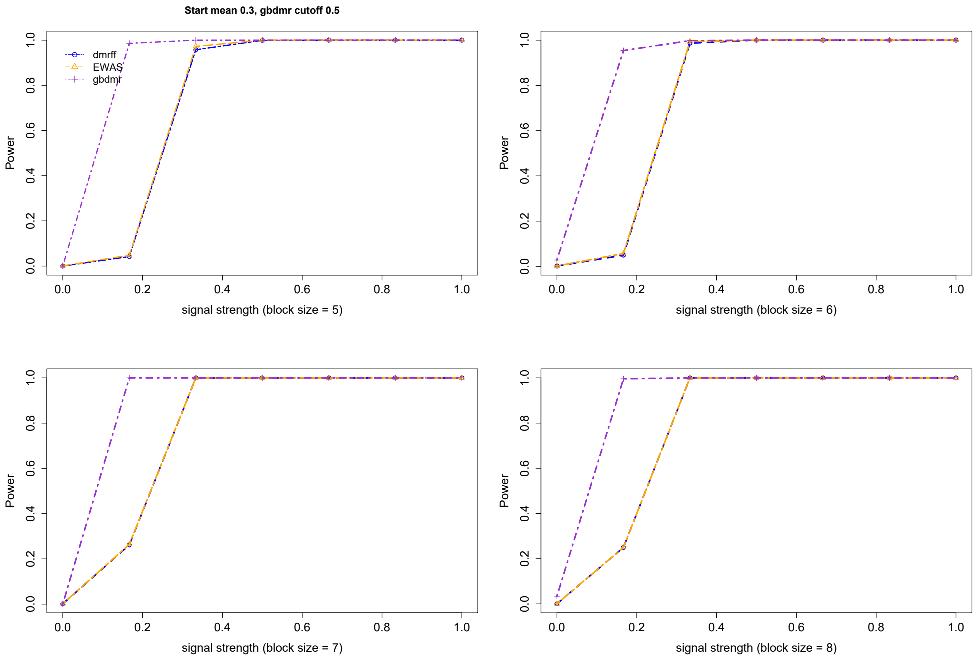
Start mean 0.3, gbdmr cutoff 0.5
dmrff
EWAS
gbdmr
Power
signal strength (block size = 5)
signal strength (block size = 6)
signal strength (block size = 7)
signal strength (block size = 8)

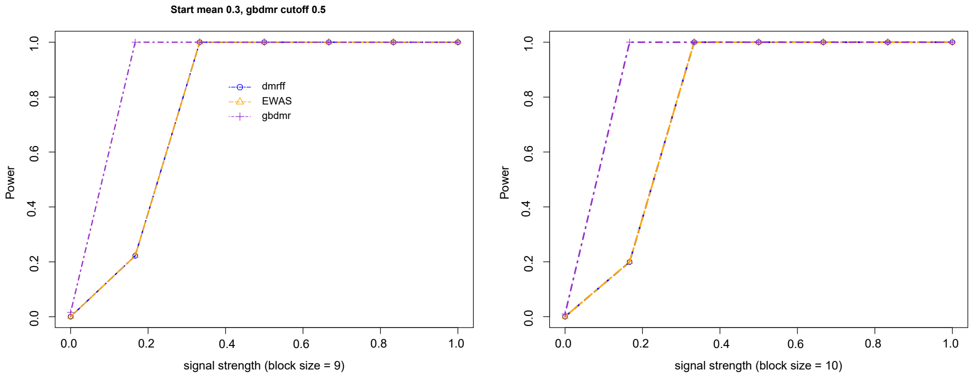
Start mean 0.3, gbdmr cutoff 0.5
dmrff
EWAS
gbdmr
Power
signal strength (block size = 9)
signal strength (block size = 10)

When the average DNAm starts from 0.5:

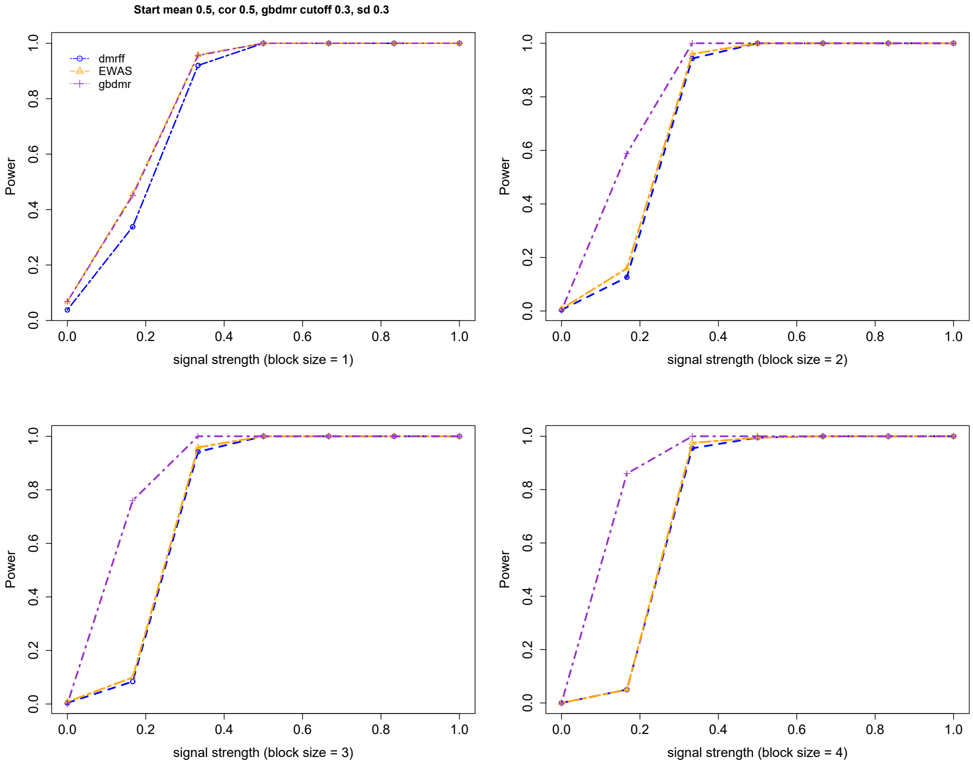

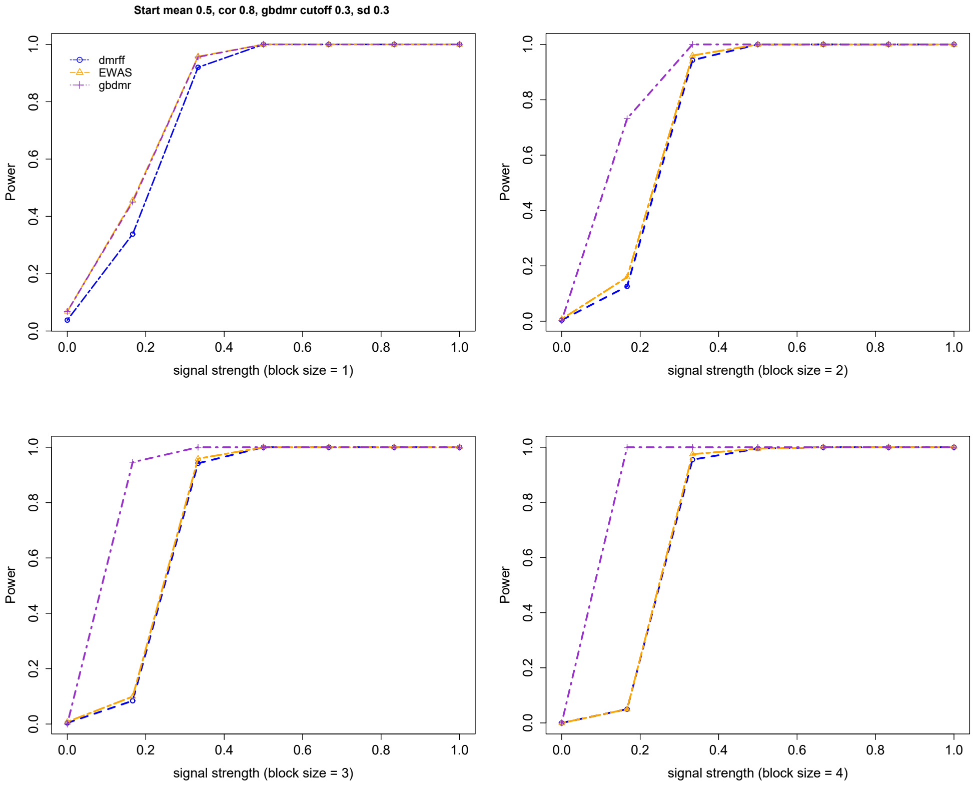
Start mean 0.5, cor 0.8, gbdmr cutoff 0.3, sd 0.3
dmrff
EWAS
gbdmr
Power
signal strength (block size = 1)
signal strength (block size = 2)
signal strength (block size = 3)
signal strength (block size = 4)

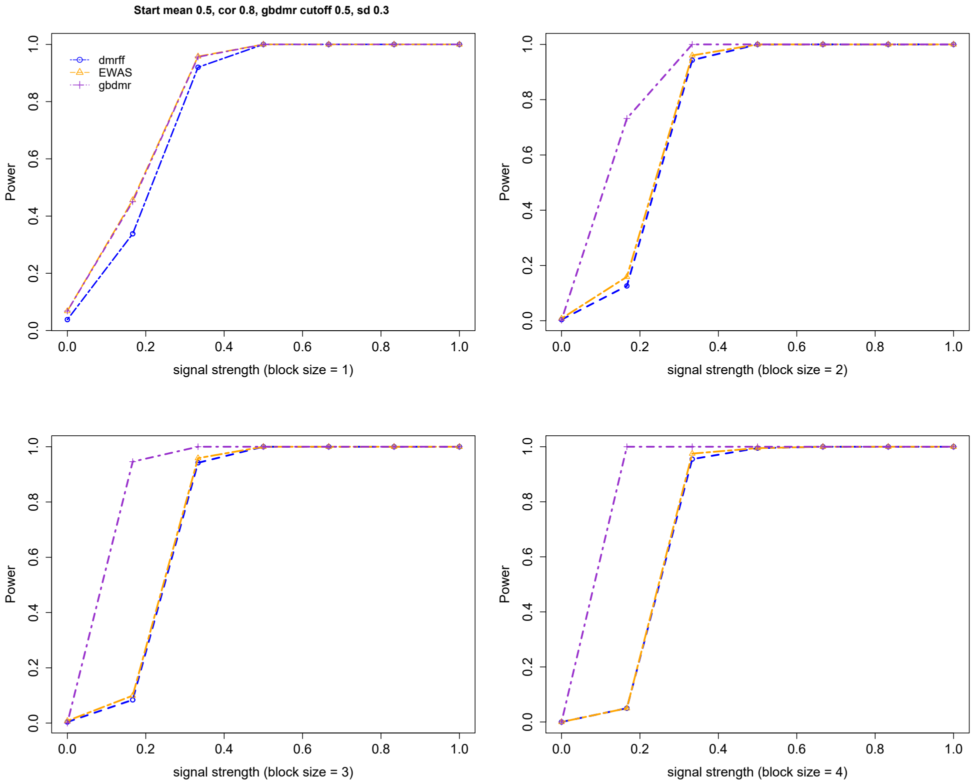
Start mean 0.5, cor 0.8, gbdmr cutoff 0.5, sd 0.3
dmrff
EWAS
gbdmr
Power
signal strength (block size = 1)
signal strength (block size = 2)
signal strength (block size = 3)
signal strength (block size = 4)

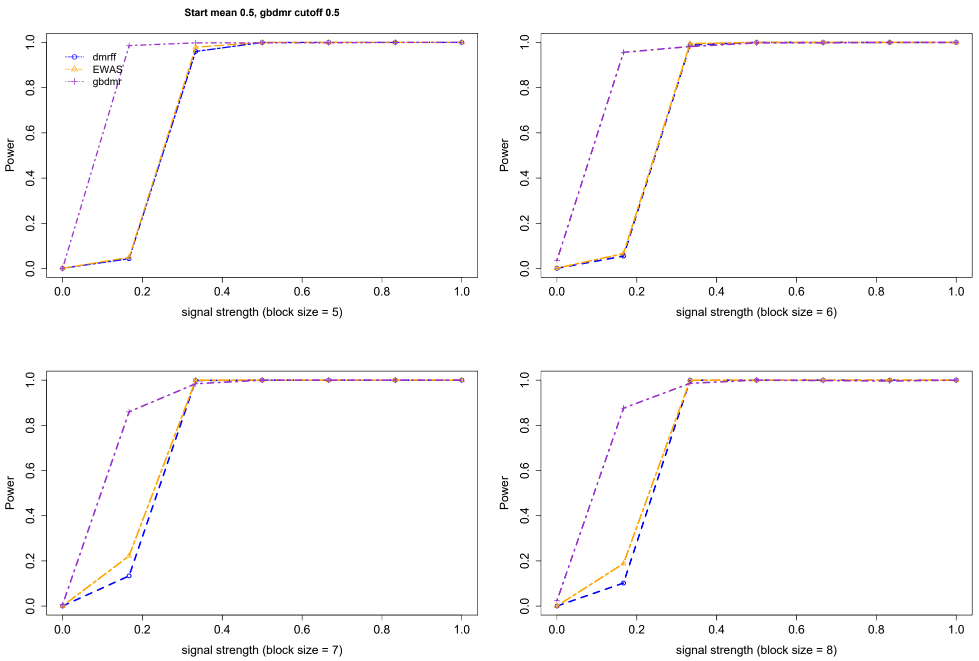
Start mean 0.5, gbdmr cutoff 0.5
dmrff
EWAS
gbdmr
Power
signal strength (block size = 5)
signal strength (block size = 6)
signal strength (block size = 7)
signal strength (block size = 8)

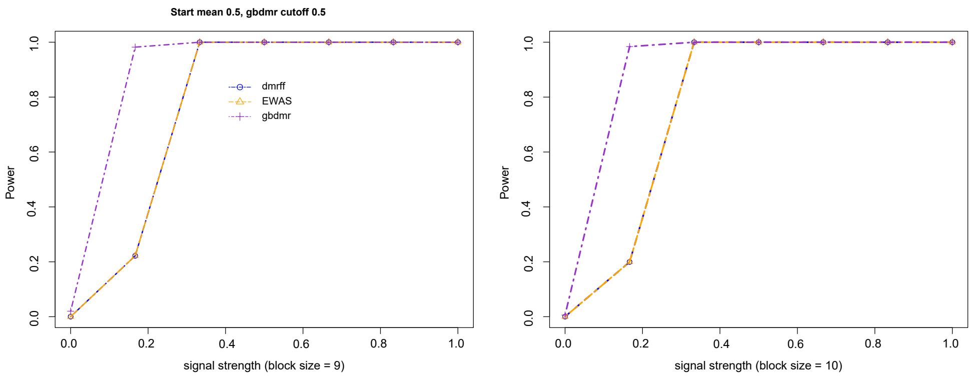
Start mean 0.5, gbdmr cutoff 0.5
dmrff
EWAS
gbdmr
Power
signal strength (block size = 9)
signal strength (block size = 10)

When the average DNAm starts from 0.7:

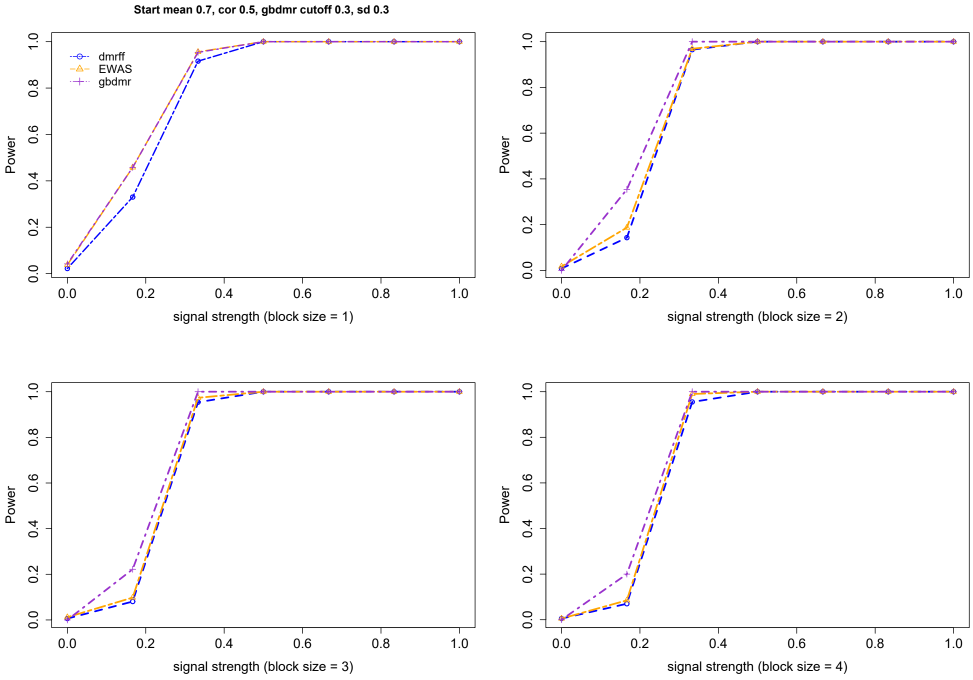

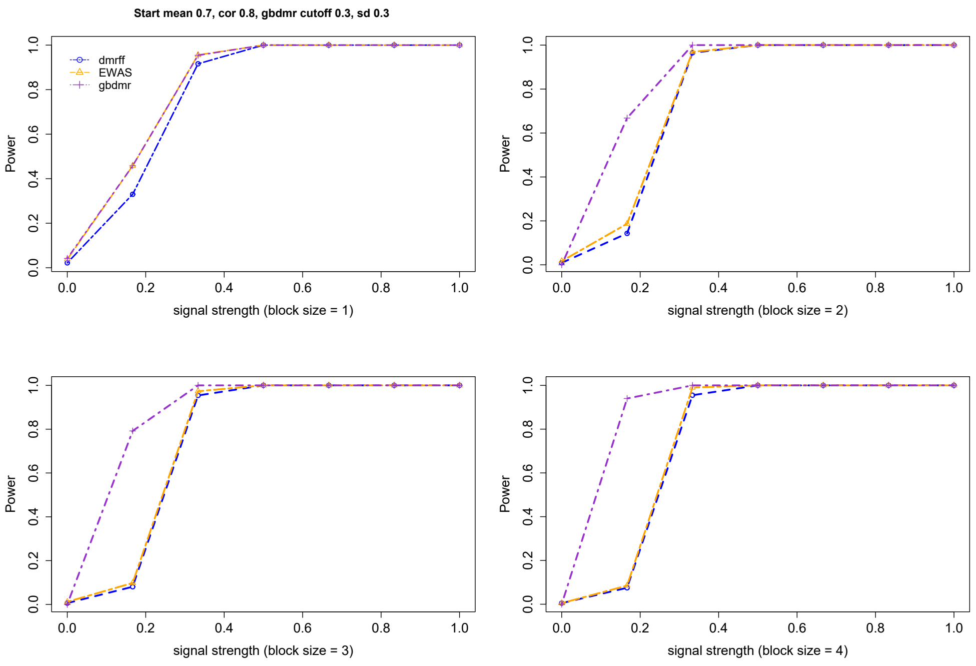
Start mean 0.7, cor 0.8, gbdmr cutoff 0.3, sd 0.3
dmrff
EWAS
gbdmr
Power
signal strength (block size = 1)
signal strength (block size = 2)
signal strength (block size = 3)
signal strength (block size = 4)

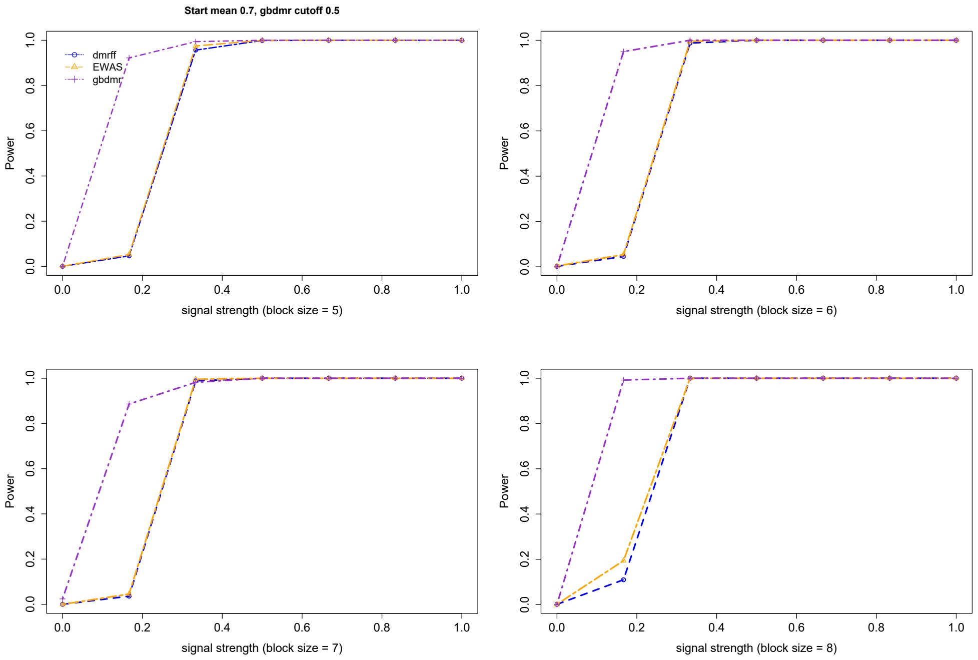
Start mean 0.7, gbdmr cutoff 0.5
dmrff
EWAS
gbdmr
Power
signal strength (block size = 5)
signal strength (block size = 6)
signal strength (block size = 7)
signal strength (block size = 8)

Start mean 0.7, gbdmr cutoff 0.5

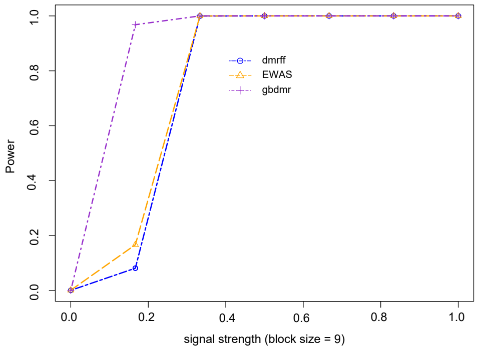

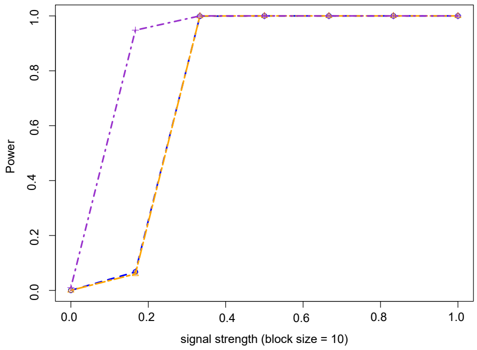

Appendix C

Figure 4 of the main paper presents the Venn plot of CpG sites (DMP CpGs + DMR CpGs) identified by EWAS, dmrff, and gbdmr. Appendix C presents the Venn plot of DMP CpGs only.

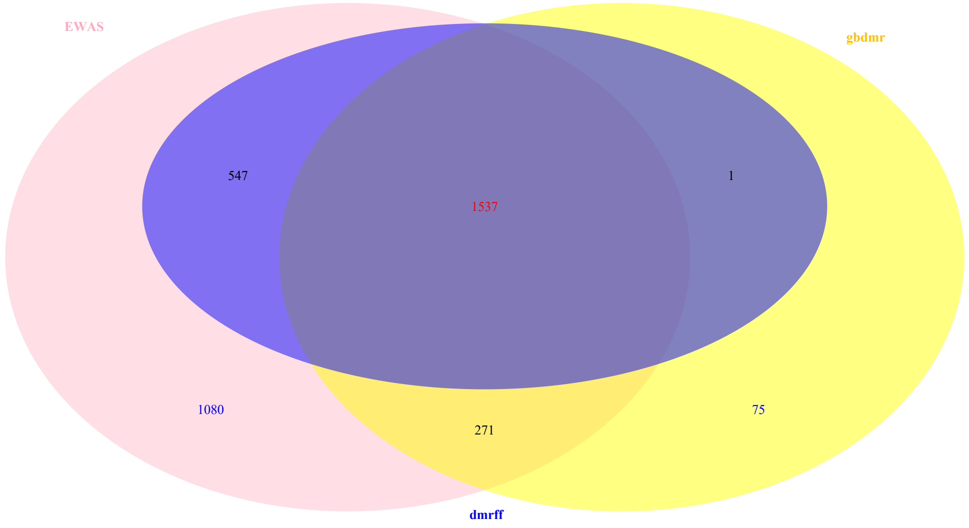

Appendix D

In Appendix D, we recorded the execution time and memory usage of various methods for different sample sizes and CpG numbers. The gbdmr method was implemented using Intel Skylake Gold 6148 Processors (core frequency: 2.40 GHz) with the processing distributed across 8 parallel tasks. Table 1 lists the results based on 346k CpGs with sample sizes ranging from 50 to 500, and Table 2 is for a fixed sample size of 200 but varying numbers of CpG sites ranging from 100k and 350k.

Table 1 Appendix D.1

	Time (Min)			Memory (Mb)		
Sample size	dmrff	EWAS	gbdmr	dmrff	EWAS	gbdmr
50	1	18	22	687	463	1380
100	1.2	20	26	970	883	1660
150	1.2	22	30	1250	1302	1930
200	1.5	25	34	1527	1723	2210
250	1.5	27	38	1810	2143	2490
300	1.6	27	42	2090	2562	2760
400	1.6	28	51	2647	3402	3320
500	1.7	29	60	3207	4242	3870

Fix the CpG numbers as 346k; sample size ranges from 50-500. A parallel computation algorithm has been implemented in gbdmr, and the data processing is carried out with eight parallel tasks.

Table 2 Appendix D.2

	Time (Min)			Memory (Mb)		
CpG numbers (K)	dmrff	EWAS	gbdmr	dmrff	EWAS	gbdmr
100	0.6	3	11	637	492	1310
150	0.7	7	15	815	738	1490
200	0.9	15	19	993	984	1680
250	0.9	16	24	1171	1230	1860
300	0.9	16	29	1349	1476	2040
350	1	19	35	1527	1722	2220

Fix the sample size as 200; CpG number ranges from 100-350K. A parallel computation algorithm has been implemented in gbdmr, and the data processing is carried out with eight parallel tasks.

Appendix E

In Appendix E, we revisited the key simulations from Appendices A and B to assess the three methods under an unbalanced design. To this end, we altered the simulation parameters, setting the DNAm at 422/84 instead of the equal 253/253 distribution for a binary exposure such as disease.

Power and false positive rate by correlation strengths of adjacent CpGs:

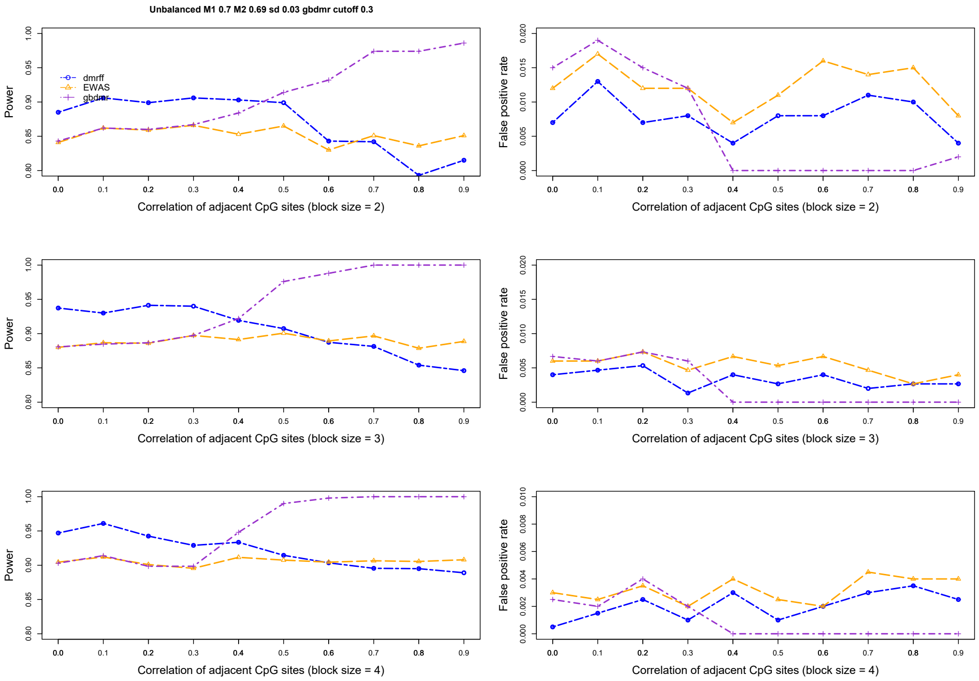

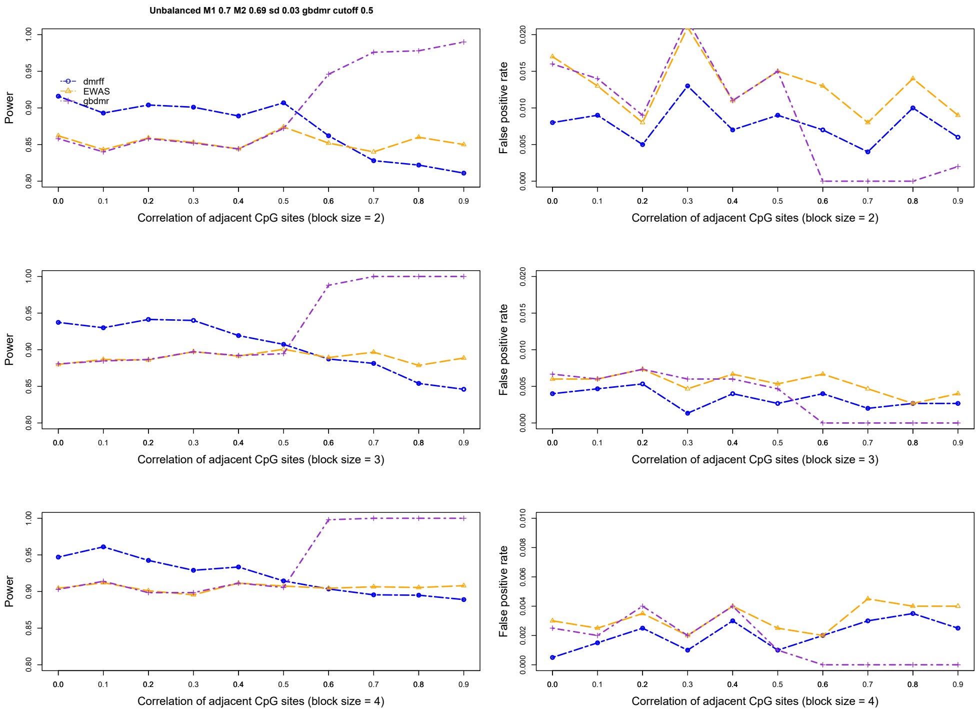

Unbalanced M1 0.7 M2 0.69 sd 0.03 gbdmr cutoff 0.5
dmrff
EWAS
gbdmr
Power
False positive rate
Correlation of adjacent CpG sites (block size = 2)
Correlation of adjacent CpG sites (block size = 3)
Correlation of adjacent CpG sites (block size = 4)

Power by signal strength:

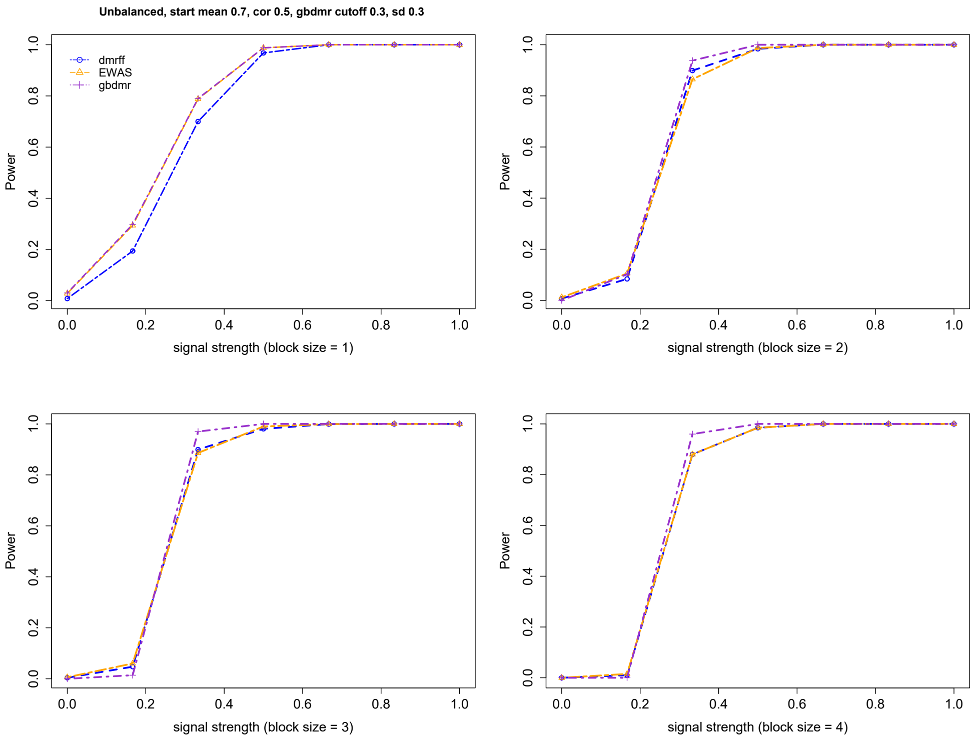

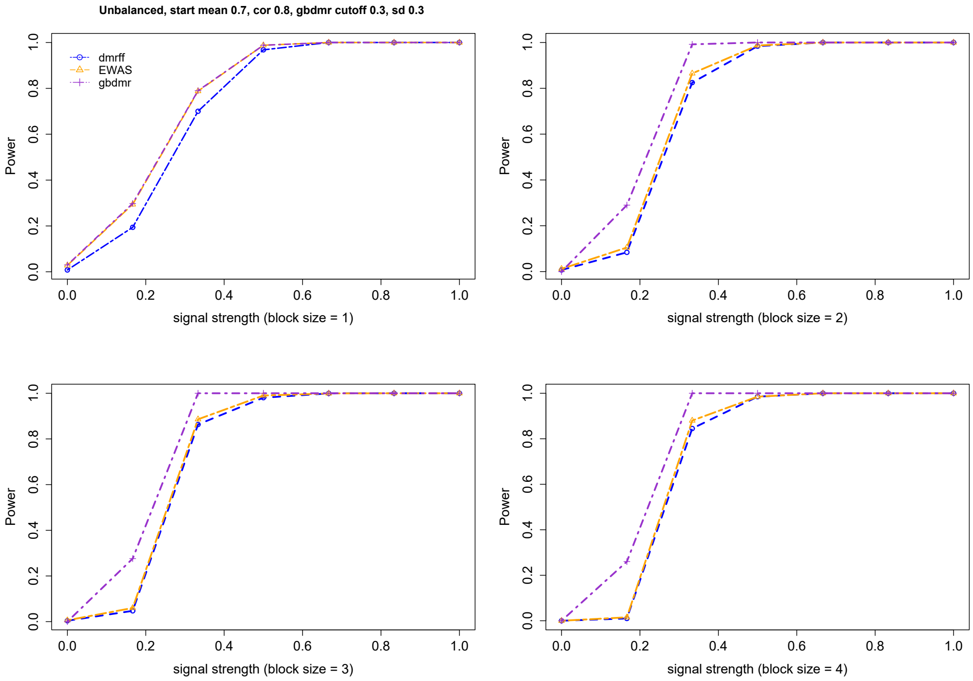
Unbalanced, start mean 0.7, cor 0.8, gbdmr cutoff 0.3, sd 0.3
dmrff
EWAS
gbdmr
Power
signal strength (block size = 1)
signal strength (block size = 2)
signal strength (block size = 3)
signal strength (block size = 4)

Appendix F

In Appendix F, we repeated the key simulations from Appendices A and B using a continuous phenotype instead of a binary phenotype. Specifically, we first simulated the DNAm following gamma distribution such that the mean equals μ and standard deviation equals σ. The σ is fixed, and the μ's varied across an evenly spaced grid ranging from 0.69 to 0.70. For each μ level, we generated the corresponding continuous phenotype using a normal distribution with the same mean (μ) and a standard deviation of 0.002. To generate blocks of size > 1, we first simulated the DNAm of a single CpG site following beta distributions. Then, we generated a second vector such that it has a fixed correlation with the first CpG site with the same mean and standard deviation. We followed the same step to generate the third CpG site's DNAm given the second, and so on. This procedure was used to simulate a chain of CpG sites with a given correlation between adjacent CpG sites. Our analysis includes the assessment of power and false positive rate at different correlation strengths of adjacent CpGs (as in Appendix A.2) and power at different signal strengths (as in Appendix B). We examined the block sizes varying from 1 to 10, and all simulations are repeated 500 times.

Power and false positive rate by correlation strengths of adjacent CpGs:

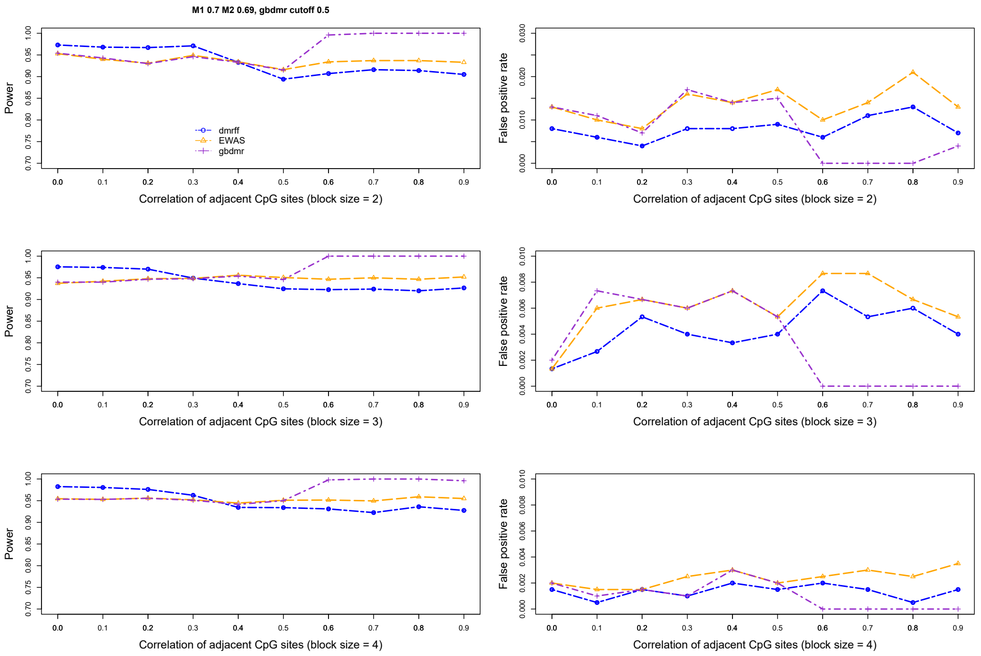

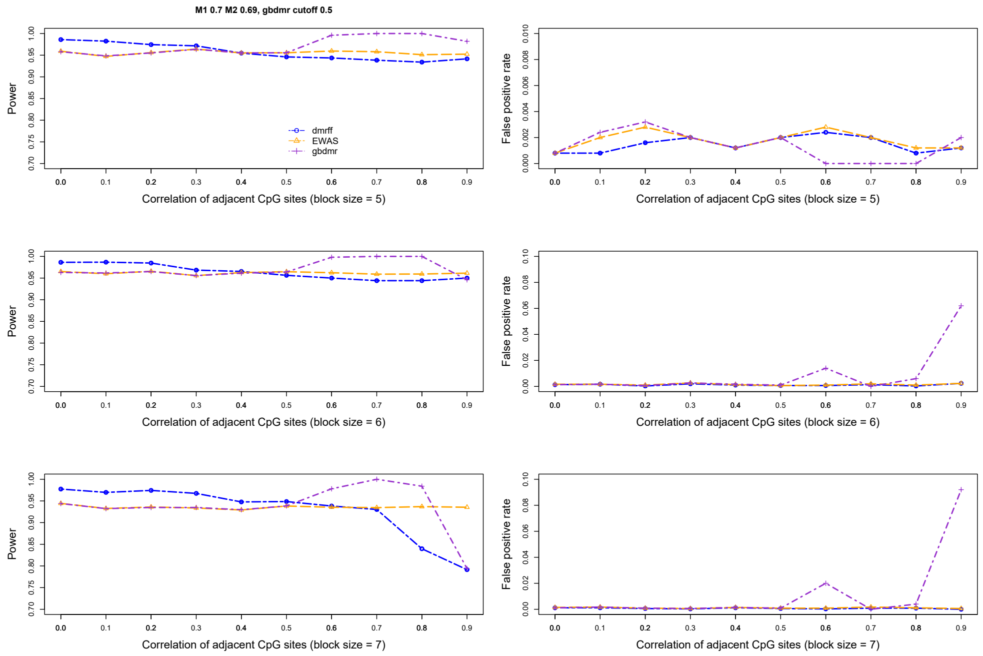
M1 0.7 M2 0.69, gbdmr cutoff 0.5
Power
False positive rate
dmrff
EWAS
gbdmr
Correlation of adjacent CpG sites (block size = 5)
Correlation of adjacent CpG sites (block size = 6)
Correlation of adjacent CpG sites (block size = 7)

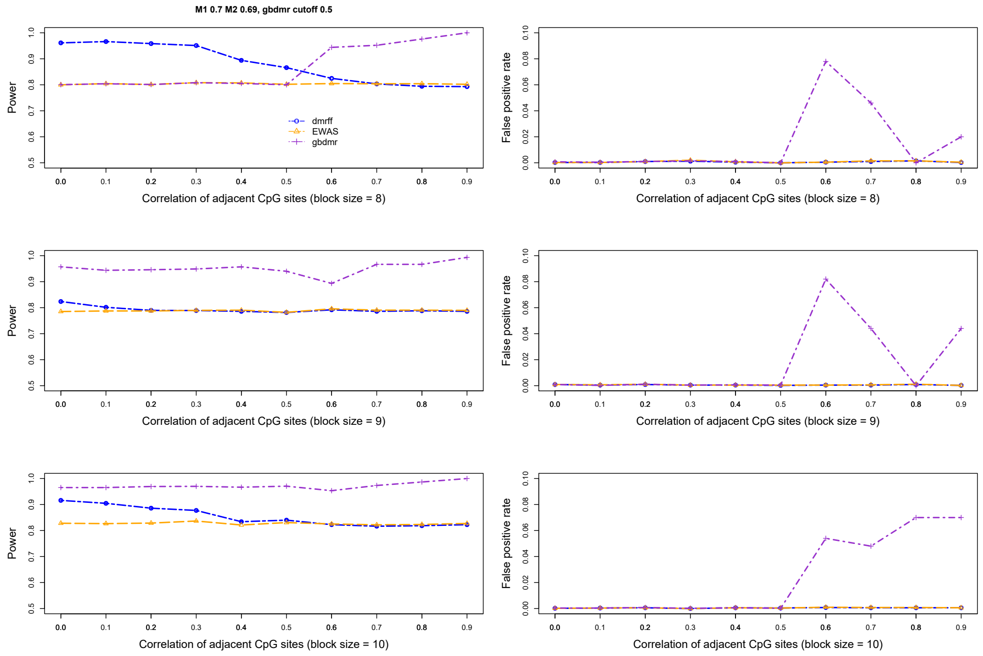
M1 0.7 M2 0.69, gbdmr cutoff 0.5
Power
False positive rate
dmrff
EWAS
gbdmr
Correlation of adjacent CpG sites (block size = 8)
Correlation of adjacent CpG sites (block size = 9)
Correlation of adjacent CpG sites (block size = 10)

Power by signal strength:

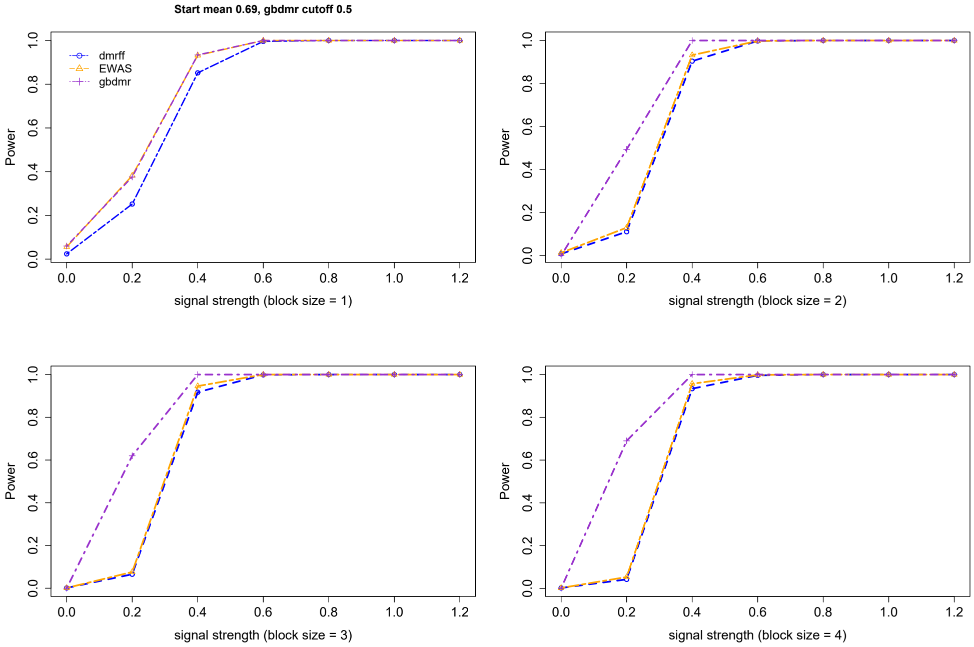

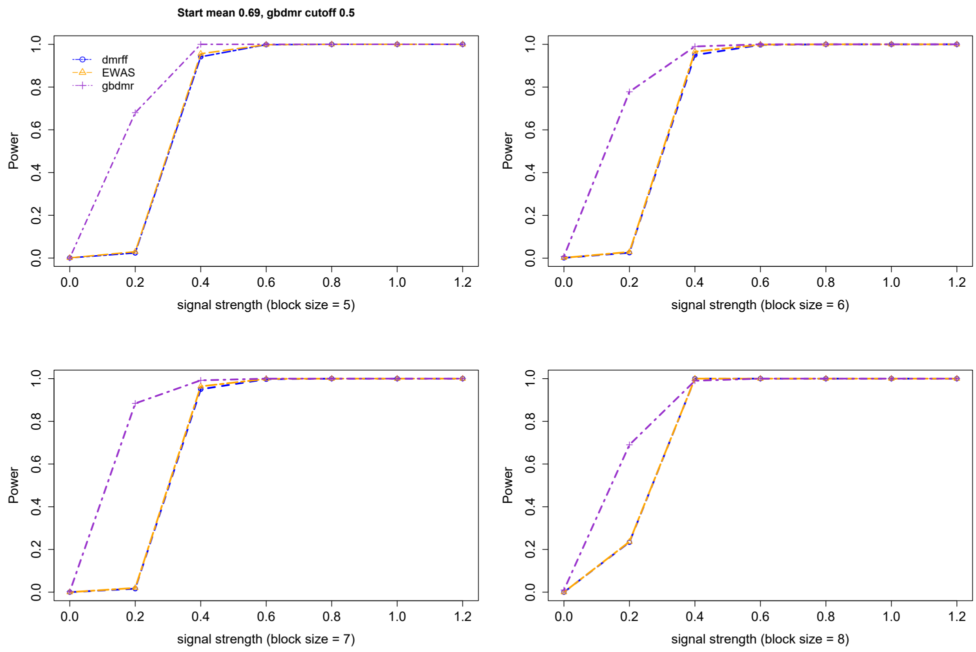
Start mean 0.69, gbdmr cutoff 0.5
dmrff
EWAS
gbdmr
Power
signal strength (block size = 5)
signal strength (block size = 6)
signal strength (block size = 7)
signal strength (block size = 8)

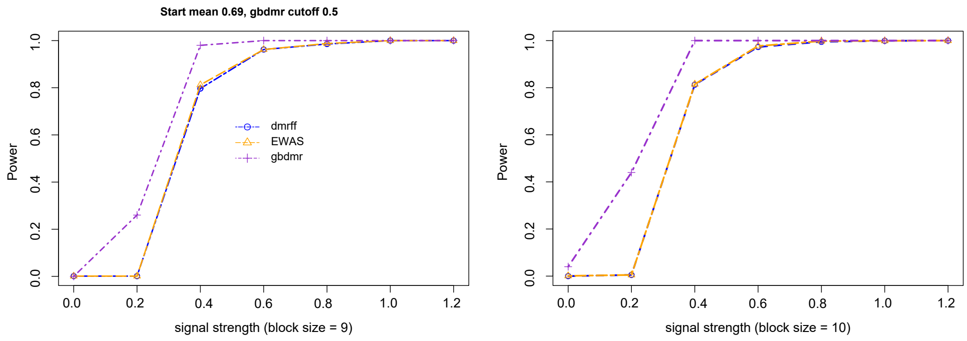
Start mean 0.69, gbdmr cutoff 0.5
dmrff
EWAS
gbdmr
Power
signal strength (block size = 9)
signal strength (block size = 10)

Appendix G

Technical details in the power of dmrff:

Let

$$Y_{ij} = \mu_j + X_i\beta + \epsilon_{ij}$$

denote the DNA methylation (DNAm) levels of jth CpG site of the ith sample, where $i = 1, \ldots, n$; $j = 1, \ldots, L_b$ (assuming the bth block of CpG sites consist of L_b CpG sites). We assume the phenotype X_i's have the same effect β on L_b CpG sites in the differentiated DNAm region (DMR), and ϵ_{ij} and $\epsilon_{ij'}$ $(j \neq j')$ are correlated, with $(\epsilon_{i1}, \ldots, \epsilon_{iL_b})^\top \sim MVN(\mathbf{0}, \mathbf{\Sigma})$, where $\mathbf{0} = (0, \ldots, 0)^\top$, and

$$\mathbf{\Sigma} = \sigma^2 \begin{bmatrix} 1 & \rho & \ldots & \rho \\ \rho & 1 & \ldots & \rho \\ \vdots & \vdots & \ddots & \rho \\ \rho & \rho & \rho & 1 \end{bmatrix}.$$

The estimate of β using the jth CpG site's DNA mehtylation, $\widehat{\beta}_j = e_2(\mathbf{X}^\top\mathbf{X})^{-1}\mathbf{X}^\top\mathbf{Y}_j$, where $e_2 = (0, 1)^\top$,

$$\mathbf{X} = \begin{bmatrix} 1 & X_1 \\ \vdots & \\ 1 & X_n \end{bmatrix}.$$

and $\mathbf{Y}_j = (Y_{1j}, \ldots, Y_{nj})^\top$. Following these notations, $\widehat{\beta}_j$ is an ordinary linear regression estimator with $E(\widehat{\beta}_j) = \beta$. The covariance between $\widehat{\beta}_j$ and $\widehat{\beta}_{j'}$,

$$\text{cov}(\widehat{\beta}_j, \widehat{\beta}_{j'}|\mathbf{X}) = \begin{cases} \sigma_n^2, & \text{if } j = j' \\ \rho\sigma_n^2, & \text{if } j \neq j', \end{cases}$$

where $\sigma_n^2 = \sigma^2 e_2 \top (\mathbf{X}^\top\mathbf{X})^{-1} e_2 = \sigma^2 / \sum_{i=1}^n (X_i - \bar{X})^2$ and $\bar{X} = \sum_{i=1}^n X_i/n$. Thus, we have the distribution of $\widehat{\boldsymbol{\beta}} = (\widehat{\beta}_1, \ldots, \widehat{\beta}_{L_b})^\top$ follows a multivariate normal distribution with mean $\boldsymbol{\beta} = (\beta, \ldots, \beta)^\top$ and covariance matrix

$$\mathbf{\Omega} = \sigma_n^2 \begin{bmatrix} 1 & \rho & \ldots & \rho \\ \rho & 1 & \ldots & \rho \\ \vdots & \vdots & \ddots & \rho \\ \rho & \rho & \rho & 1 \end{bmatrix}.$$

Denote by $A = (\mathbf{1}^\top\mathbf{\Omega}^{-1}\mathbf{1})^{-1}\mathbf{1}^\top\mathbf{\Omega}^{-1}\widehat{\boldsymbol{\beta}}$. Then $E(A|\boldsymbol{X}) = (\mathbf{1}^\top\mathbf{\Omega}^{-1}\mathbf{1})^{-1}\mathbf{1}^\top\mathbf{\Omega}^{-1}\mathbf{1}\beta = \beta$ and $\text{var}(A|\boldsymbol{X}) = (\mathbf{1}^\top\mathbf{\Omega}^{-1}\mathbf{1})^{-1}\mathbf{1}^\top\mathbf{\Omega}^{-1}\mathbf{\Omega}\mathbf{\Omega}^{-1}\mathbf{1}(\mathbf{1}^\top\mathbf{\Omega}^{-1}\mathbf{1})^{-1} = (\mathbf{1}^\top\mathbf{\Omega}^{-1}\mathbf{1})^{-1}$. Note that the σ in

Ω, the true standard deviation of the error term, is not known. Dmrff estimates Ω by

$$\widehat{\boldsymbol{\Omega}} = \widehat{\boldsymbol{\sigma}}\widehat{\boldsymbol{\sigma}}^\top \odot \begin{bmatrix} 1 & \rho & \dots & \rho \\ \rho & 1 & \dots & \rho \\ \vdots & \vdots & \ddots & \rho \\ \rho & \rho & \rho & 1 \end{bmatrix},$$

where $\widehat{\boldsymbol{\sigma}} = (\widehat{\sigma}_1, \dots, \widehat{\sigma}_{L_b})^\top$, $\widehat{\sigma}_j$ is the standard error of $\widehat{\beta}_j$, and $\odot$ is the element-wise multiplication of matrix. For simplicity, we assume σ_n is known, and thus the estimator of dmrff is expressed as $A/\sqrt{\text{var}(A|\boldsymbol{X})}$. To derive its power, note that

$$\frac{A}{\sqrt{\text{var}(A|\boldsymbol{X})}} = \frac{A-\beta}{\sqrt{\text{var}(A|\boldsymbol{X})}} + \frac{\beta}{\sqrt{\text{var}(A|\boldsymbol{X})}}.$$

Thus, the power of dmrff is

$$P(\frac{A-\beta}{\sqrt{\text{var}(A|\boldsymbol{X})}} > Z_{1-\alpha/2} - \frac{\beta}{\sqrt{\text{var}(A|\boldsymbol{X})}}) + P(\frac{A-\beta}{\sqrt{\text{var}(A|\boldsymbol{X})}} < Z_{\alpha/2} - \frac{\beta}{\sqrt{\text{var}(A|\boldsymbol{X})}})$$
